# ﻿An integrative approach to the delimitation of pseudocryptic species in the *Eucyclopssperatus* complex (Copepoda, Cyclopoida) with a description of a new species

**DOI:** 10.3897/zookeys.1226.138389

**Published:** 2025-02-07

**Authors:** Aleksandr Novikov, Dayana Sharafutdinova, Ekaterina Abramova, Tatyana Mayor, Elena Chertoprud

**Affiliations:** 1 A.N. Severtsov Institute of Ecology and Evolution, Leninsky Prospect 33, Moscow 119071, Russia A.N. Severtsov Institute of Ecology and Evolution Moscow Russia; 2 Kazan Federal University, Kremlyovskaya St. 18, Kazan 420008, Russia Kazan Federal University Kazan Russia; 3 Lena Delta Nature Reserve, Tiksi, 678400 Russia Lena Delta Nature Reserve Tiksi Russia; 4 Limnological Institute, Siberian Branch of the Russian Academy of Sciences, 3 Ulan-Batorskaya St., Irkutsk 664033, Russia Siberian Branch of the Russian Academy of Sciences Irkutsk Russia

**Keywords:** Biodiversity, cryptic species, cuticular pores, Cyclopidae, *Eucyclopssibiricus*, geometric morphometrics, integrative description, linear morphometrics

## Abstract

*Eucyclopssperatus* (Lilljeborg, 1901) (Copepoda, Cyclopoida, Cyclopidae) was studied using various methods. Molecular genetic methods (comparison of COI and ITSn molecular markers) have shown that this species represents a species complex, and the following methods were used to search for differences between the species: analysis of qualitative and quantitative characters, linear morphometrics, landmark-based geometric morphometrics, and integumental pore pattern of the cephalothorax. *Eucyclopssibiricus***sp. nov.** from Middle Siberia is described. The two studied species can be considered pseudocryptic; the main morphological difference between the species is the number of setules on the inner side of the first and second exopod segments of the fourth pair of swimming legs: *E.sibiricus***sp. nov.** has 6–10 and 7–17 setules, respectively; *E.speratus* has 0–3 and 0–6 setules, respectively. The morphometry and integumental pore pattern of the cephalothorax were ineffective for identification and separation of species. The existing previous records of *E.speratus* were also analyzed, and the records of this species in the Irkutsk region (Russia), as well as in Japan and Korea, are attributed to *E.sibiricus***sp. nov.**

## ﻿Introduction

In the present paper, we continue our systematic study describing the diversity and fauna of Copepoda in Siberia (Novikov et al. 2024). This time our attention was focused on the genus *Eucyclops* Claus, 1893 from the family Cyclopidae Rafinesque, 1815. This genus is probably one of the most difficult to identify and study taxonomically (Mercado-Salas et al. 2016) for several reasons:

- It includes > 100 valid species according to Alekseev (2019) and ~ 150 species and subspecies according to our calculations.
- The species of the genus are morphologically uniform (Mercado-Salas et al. 2016), have a constant number of segments, setae, and spines on the antennules, mouthparts, and swimming legs, except for the symbiotic *E.bathanalicola* Boxshall & Strong, 2006 with reduced mouthparts (Boxshall and Strong 2006).
- The complex genetic structure of some species is shown, for example, in *E.serrulatus* Fischer, 1851 (Hamrová et al. 2012; Sukhikh and Alekseev 2015).
- The identification keys are inaccurate. Only recently the first almost complete key for identifying *Eucyclops* of the world fauna was provided (Alekseev 2019).
- Because of the uniform morphology, the so-called micromorphological characters (Alekseev et al. 2006) are important for the identification (for example, the ornamentation of the coxobasis of the antenna 2) – those characters can be observed only upon careful examination.

The combination of the importance of “microcharacters” for identification and the fact that many species have not been described in sufficient detail (especially species described before the middle of the last century) makes even the existing keys ineffective. Therefore, we collected *Eucyclops* species from Russia to have enough individuals to analyze with different methods, including the use of genetic methods and to search for new important characters.

*Eucyclopssperatus* is a widespread species in the Palearctic. It was first described from Sweden (Lilljeborg 1901) and has been reported from Europe (Gurney 1933; Monchenko 1974), North Africa (Dumont and Decraemer 1977), Central Siberia (Chertoprud et al. 2022), Kamchatka (Ishida 1998), and Japan (Ishida and Hiruta 1999). It has probably been frequently misidentified as *E.serrulatus* (Monchenko 1974; Alekseev et al. 2006). *Eucyclopssperatus* is absent from the Nearctic (Dussart and Fernando 1990), and the presumed records from North America were first attributed to *E.neomacruroides* Dussart & Fernando, 1990 by the same authors; lately, this latter species was shown to be a junior synonym of *E.elegans* (Herrick, 1884) by Hudson et al. (2003). *Eucyclopssperatus* is possibly found only in Alaska (Ishida 1998). In a recent revision of the genus *Eucyclops*, Alekseev (2019) assigned this species to the subgenus E. (Speratocyclops) Alekseev, 2019.

Our first collection of *E.speratus* from Siberia was in the Lena River Delta in 2018 and in Putorana Plateau in 2021. The initial biomolecular analysis, in which we sequenced the mitochondrial COI and nuclear ITSn genes of *E.speratus* individuals from the Lena Delta and the Putorana Plateau showed that these populations were genetically different from the supposed conspecifics from the vicinity of St. Petersburg, the European part of Russia. This raised several questions:

1. Are populations from Siberia and the European part of Russia different species, different geographic subspecies, or do the differences reflect a genetic variability within a single species?
2. Do the populations of Siberia and Europe differ morphologically, and if so, to what extent? Are they well differentiated, or can they be considered pseudocryptic?
3. If there are morphological differences, what characters can be used for identification?

To answer the second and third questions, we chose analyses based on qualitative characters (presence or absence of groups of spinules), quantitative characters (number of spinules and setules in groups of spinules), linear morphometrics, landmark-based geometric morphometrics, and integumental pore pattern of the cephalothorax.

## ﻿Materials and methods

### ﻿Materials

**Figure 1.**
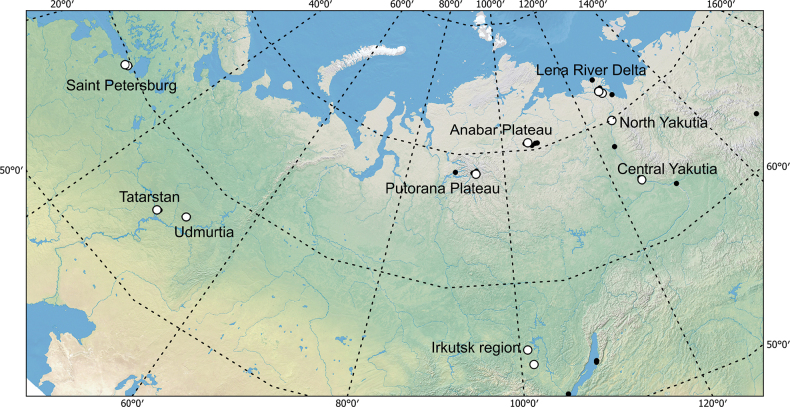
Map of sample collections with representatives of the *E.speratus* group. White dots – *E.speratus* group; black dots – other *Eucyclops* species.

Sampling was conducted at various locations in Russia, from 2018 to 2023. Small plankton nets with a mesh size of 80–100 μm were used for collection. In lakes, ponds, and puddles, samples were collected from the shore by filtering the water column and sweeping the bottom substrate. In rivers, a net was deployed in the course of a river or stream for 30 min to collect drifting organisms. Samples were fixed in 4% formalin or 96% ethanol. Individuals of the *E.speratus* species group from 16 samples were used for analysis (Table 1, Fig. 1). Biomolecular and morphological analysis were conducted for different individuals from the same population and for all the available populations. The material was deposited in the Zoological Museum of Kazan Federal University (**EMKSU**), in the Limnological Institute SB RAS (**LI SB RAS**) and in the authors’ personal collections.

**Table 1.** List of samples containing representatives of the *E.speratus* group; numbers and type of analysis for each studied individual.

Location	n	Coordinates (°N, °E)	Date	Type of waterbody	Collector	Studied individuals/type of analysis
Lena River Delta	1	72.38755, 126.48205	08.09.2020	Floodplain puddle	Abramova E.	Morphology № 45, 47, 48, 49
	2	72.38755, 126.48205	18.07.2021	Floodplain puddle	Novikov A.	Morphology № 17, 19; Genetic № ch5, ch6, ch7, ch8
	3	72.36874, 126.47595	20.08.2019	Floodplain puddle	Novikov A.	Morphology № 27, 88; Pore pattern
	4	72.11877, 126.98782	22.07.2021	Small river	Novikov A.	Morphology № 21
North Yakutia	5	69.97000, 125.60158	28.06.2019	Lake	Novikov A.	Morphology № 89, 90, 91
Central Yakutia	6	64.94187, 124.84179	22.06.2019	Temporary puddles	Novikov A.	Morphology № 28, 69, 73
Anabar Plateau	7	70.64073, 105.90506	25.07.2023	Small lake	Novikov A., Chertoprud E.	Morphology № 79
Irkutsk region	8	54.34722, 101.52861	10.06.2021	Pond	Dzyba G.	Genetic № K1-4
	9	55.3905, 100.8231	25.07.2021	River	Mayor T.	Genetic № T13
Putorana Plateau	10	68.60415, 94.05641	07.08.2021	Shallow lake	Chertoprud E.	Morphology № 32 Genetic № ch1, ch2, ch3
	11	68.60344, 94.05875	07.08.2021	Shallow lake	Chertoprud E.	Morphology № 33
	12	68.60029, 94.05300	07.08.2021	Shallow lake	Chertoprud E.	Morphology № 34
Udmurtia	13	56.86162, 52.21630	16.06.2018	Pond	Novikov A.	Morphology № 30, 59, 60, 93, 95, 96; Pore pattern
Tatarstan	14	55.76324, 48.85700	28.06.2020	Puddle near a stream	Novikov A., Sharafutdinova D.	Morphology № 75, 92, 103, 104
Saint Petersburg	15	60.01293, 30.35514	20.06.2023	Olginsky Pond	Chertoprud E.	Morphology № 97
Saint Petersburg	16	59.84890, 30.04384	20.06.2023	Orlov Pond	Chertoprud E.	Morphology № 86, 98, 111

### ﻿Molecular analysis

For DNA extraction, biological tissue (eggs, antennule, or the entire female of *Eucyclops*) was incubated in a solution comprising 2× Encyclo buffer for PCR (Eurogen, Russia) and 0.1 mg/ml Proteinase K for 1–3 h at 56 °C. The enzyme was then inactivated by heating for 5 min at 96 °C. A gene fragment of cytochrome c oxidase of mtDNA (COI), the first and second internal transcribed spacers, the 5.8S rRNA gene (ITSn), and a gene fragment of 18S rRNA of nuclear DNA were analyzed. PCR was performed using universal primers to amplify COI and ITSn and specific primers for Cyclopoida to amplify 18S rRNA, which were designed in this study (Table 2). The PCR (10 µl) contained 5–10 ng total DNA, 1× Encyclo PCR buffer, 3.5 mM magnesium, 0.5 µM of each primer, 0.2 mM of each dNTP, and 0.5 units of Encyclo DNA polymerase (Evrogen, Russia). Amplification conditions consisted of 4 min at 95 °C, and then 15 sec at 94 °C, 20 sec at annealing temperature (Table 2), 1 min at 72 °C for 35–40 cycles, and the final elongation stage for 4 min at 72 °C. The amplicons were extracted from the agarose gel for sequencing following the previously described protocol (Mayor et al. 2010). The sequences were determined be-directly using the ABI PRISM BigDye Terminator v. 3.1 sequencing kit in a Nanophor 05 genetic analyzer (Sintol, Russia). The sequences were manually edited and assembled into a consensus using UNIPRO UGENE 49.1 (Okonechnikov et al. 2012). The obtained sequences were deposited in the GenBank. Additional sequences of *Eucyclops* were obtained from GenBank and used for phylogenetic analysis. The NCBI accession numbers of these are given on the phylogenetic trees. The sequences were aligned using the MegaX (Kumar et al. 2018) and CLC Sequence Viewer 8.0.0 (https://resources.qiagenbioinformatics.com/manuals/clcsequenceviewer/current/index.php?manual=Introduction_CLC_Sequence_Viewer.html, accessed on 9 Aug 2024). The genetic distances were calculated using MegaX (Kumar et al. 2018). The nucleotide substitutions model was selected according to the Bayesian Information Criterion (BIC) using MegaX to calculate the model-corrected genetic distances. The entire lengths of ITS1 and ITS2 were determined in alignment with the KF153695.1 sequence of *Macrocyclopsdistinctus* (Richard, 1887), for which the boundaries of these spacers are known. Maximum Likelihood trees were constructed, using IQ-TREE2 (Minh et al. 2020). The trees were visualized and edited using Interactive Tree Of Life (iTOL) v. 6.8.1 (https://itol.embl.de, accessed on 9 Aug 2024) (Letunic and Bork 2021). The Basic Local Alignment Search Tool (BLAST) and Barcode of Life Data Systems v4 (BOLD) used to search for sequence similarity (Altschul et al. 1990; Ratnasingham et al. 2024). To propose species partitions from COI data sets, we used species-delimitation methods that employ pairwise genetic distances and tree-based methodologies. We used the Assemble Species by Automatic Partitioning (ASAP) tool with the p-distances and default settings (https://bioinfo.mnhn.fr/abi/public/asap/, accessed on 13 Sept 2024) (Puillandre et al. 2021) and the Bayesian Poisson Tree Processes (bPTP) (Zhang et al. 2013) with default settings and the ML-tree obtained in the study (https://species.h-its.org/ptp/, accessed on 23 Oct 2024).

**Table 2.** Features of PCR primers.

Molecular marker	Primer	Primer direction	Primer sequence (5’–3’)	Annealing temperature °C	Reference
COI	LCO-1490	Forward	GGTCAACAAATCATAAAGATATTGG	48–50	Folmer et al. 1994
	HCO-2198	Reverse	TAAACTTCAGGGTGACCAAAAAATCA		
ITSn	ITS-5	Forward	GGAAGTAAAAGTCGTAACAAGG	57	White et al. 1990
	ITS-4	Reverse	TCCTCCGCTTATTGATATGC		
IITS1	KP2	Forward	AAAAAGCTTCCGTAGGTGAACCTGCG	57	Phillips et al. 2000
	5.8S	Reverse	AGCTTGGTGCGTTCTTCATCGA		
18S	18Sfw1	Forward	GTT ATT TGA CGC GAC TGC CC	60	this study
	18Srv1	Reverse	CCA CCC ACC GAA TCG AGA AA		

### ﻿Morphological analysis and description

Each individual was dissected under a stereomicroscope, and each element (A2, P4, urosome, and the rest of the body for morphological analysis, and A1, A2, labrum, paragnaths, mouthparts, P1-P5, abdomen for descriptions) was placed under a separate cover slip on one or two slides. Rough drawings were generated from the printed photographs of each element and edited to obtain the final drawings, using the program CorelDraw.

Congo Red staining and fluorescence microscopy were used to obtain the image of the habitus (Michels and Büntzow 2010; Novikov et al. 2024). To stain the individuals, we first washed them in distilled water, and stored them in Congo red (1.5 mg per 1 ml) for 24 h. Next, each individual was transferred on a slide, mounted with glycerol, and covered with a coverslip. For observations, we used a ZEISS Axio Imager M2 microscope with filter settings: excitation BP 460–490 nm, beamsplitter FT 495 nm, emission BP 500–600 nm. With this filter, Congo Red glows yellow and chitin glows green. Photographs were taken using Z-stacking.

Nomenclature and descriptive terminology follow Huys and Boxshall (1991). The armature formulae of swimming legs are given according to Sewell (1949). Terminology and homology of maxillary structures follow Ferrari and Ivanenko (2008) and terminology of genital structures follows Cuoc and Defaye (2011). The numbering of the groups of spinules on the posterior side of the P4 coxa is given according to Einsle (1985); groups of spinules of the antennal coxobasis is given according to Alekseev et al. (2006).

The following abbreviations are used in the text: **ae** = aesthetasc; **P1–P6** = legs 1–6; **Enp1–Enp3** = first–third endopodal segment; **Exp1–Exp3** = first–third exopodal segment.

### ﻿Quantitative morphological analysis

For the quantitative morphological analysis, spinules/setules of each group on the right and left sides were counted. Average values between sides, rounded up, are listed in the tables in Suppl. material 1. The following groups were used for counting:

1. Antennal coxobasis groups N° 3, 4, 7, 8, 10, 12 (Alekseev et al. 2006);
2. Postero-medial row of spines of the first endopodal segment of the antenna;
3. Proximal row of setules of the intercoxal sclerite P4;
4. Spinules of the disto-lateral group of coxa P4, group E in Alekseev et al. (2006);
5. Spinules on the outer side of the coxal seta P4;
6. Setules on the inner side of P4 Exp1–Exp2;
7. Spinules on the posterior side of P4 Exp1;
8. Setules on the outer side of P4 Exp2–Exp3;
9. Serra of the caudal rami;
10. Setules on the outer side of the caudal seta VI.

### ﻿Linear morphometrics

**Figure 2.**
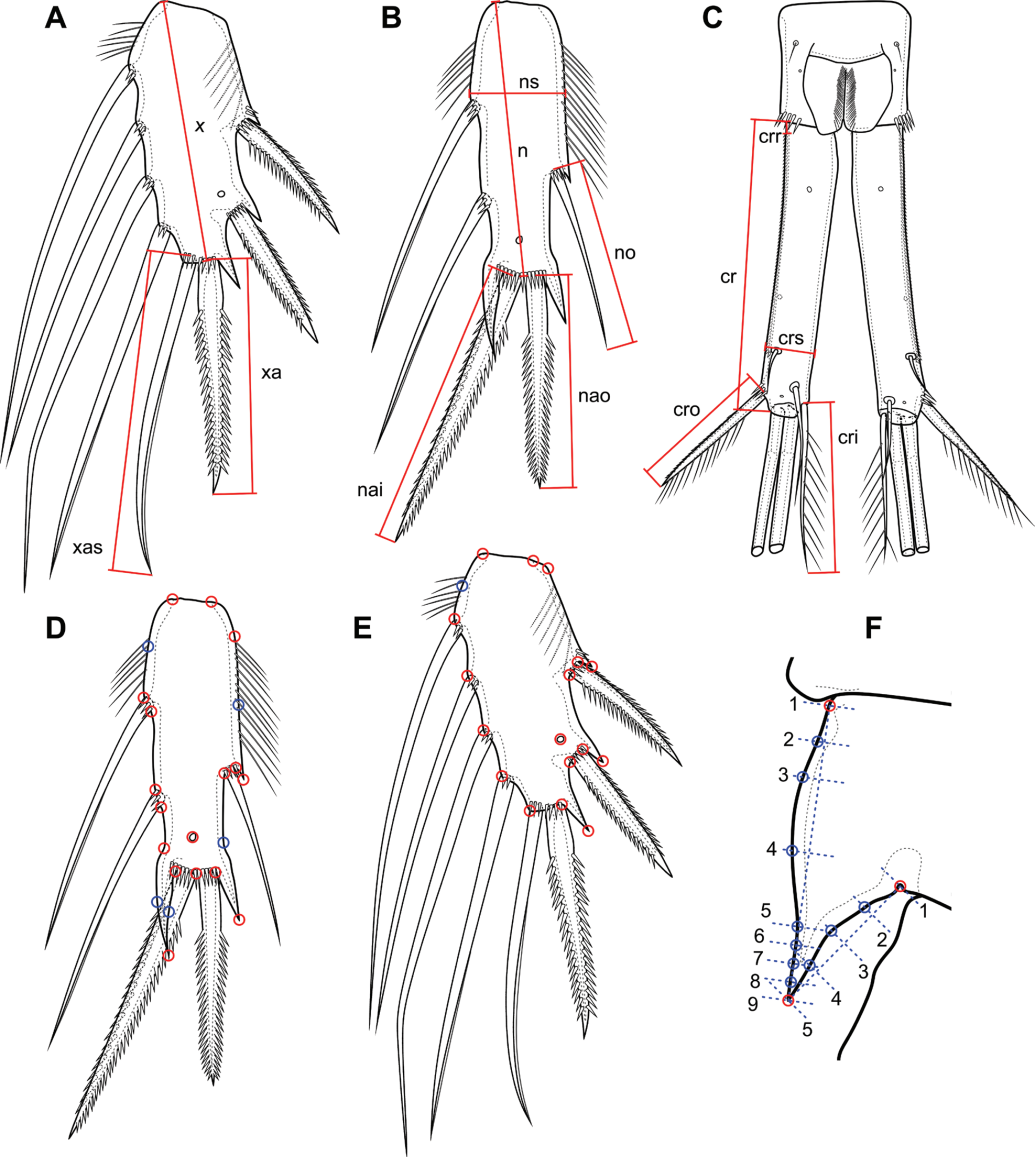
Measurement schemes for linear morphometry (**A**–**C**) and landmark arrangement schemes for geometric morphometry (**D**–**F**). **A** P4 Exp 3 **B** P4 Enp3 **C** caudal rami and anal somite **D** P4 Enp3 **E** P4 Exp 3 **F** P4 inner side of basis. Red circles are landmarks of types I and II, blue circles are semi-landmarks.

For linear morphometric analysis, the length/width values of segments and setae on the right and left sides were used (Fig. 2A–C):

1. Length of P4 Exp3 (x);
2. Length of the apical spine of P4 Exp3 (xa);
3. Length of the apical seta of P4 Exp3 (xas);
4. Length of P4 Enp3 (n);
5. Width of P4 Enp3 (ns);
6. Length of the outer seta of P4 Enp3 (no);
7. Length of the outer apical spine of P4 Enp3 (nao);
8. Length of the inner apical spine of P4 Enp3 (nai);
9. Length of the caudal rami (cr);
10. Distance from the base of the caudal rami to the first spinule of serra (crr);
11. Width of the caudal rami (crs);
12. Length of caudal seta III (cro);
13. Length of caudal seta VI (cri).

The tables in Suppl. material 1 include average values between the sides. In addition to comparing the absolute values of the measurements, the ratios between different measurements were also analyzed: xa/x; xas/x; xas/xa; n/ns; no/n; nai/nao; nai/n; cr/crs; crr/cr; cri/cro; cri/cr (Suppl. material 1).

Statistical comparisons and graphs were generated in PAST4 (Hammer and Harper 2001). Quantitative morphological data and linear morphometrics were analyzed using non-metric multidimensional scaling (nMDS) (Mead 1992) based on Gower’s distance (Gower 1971).

### ﻿Geometric morphometrics

Geometric morphometrics has been shown to be effective in the taxonomy based on rigid elements of various animals (Viacava et al. 2023), including representatives of the Cyclopidae (Karanovic and Bláha 2019). Three elements, namely the P4 Exp3, the P4 Enp3 (Karanovic and Bláha 2019), and the shape of the inner part of the basis of P4 – species-specific for some groups of Cyclopidae (Stoch 2001; Novikov et al. 2024) – were selected for geometric morphometrics.

Landmarks of P4 Exp3, P4 Enp3 (Fig. 2D, E) are divided into three main types according to Bookstein (1997): type I (discreet juxtapositions of tissues), type II (maxima of curvature) and semi-landmarks located between two existing landmarks of type I or II (Vasil’ev et al. 2018). On the inner part of the basis P4 there are three landmarks of types I and II, the remainder are semi-landmarks (Fig. 2F). To place the landmarks, photographs of each element were taken from the right and left sides with different focal lengths. Then, photographs with different focal lengths were combined with the Helicon Focus program. Landmarks were installed in the tpsDig2 program (Rohlf 2006). For the inner part of the P4 basis, before installing the semi-landmarks, lines with segments at certain distances were drawn on the photographs; the starting and ending points of these segments are the base of the inner side of the basis, the apex of the spinous process, and the bend on near the base of the endopod. On the outer side, there are three intermediate points at a distance of 25%, 50%, and 75%. On the inner side, there are seven intermediate points: 12.5% (2), 25% (3), 50% (4), 75% (5), 81.21% (6), 87.5% (7), and 93.75% (8). In the distal part, there are more points for a more accurate description of the shape of the spinous process. Next, landmarks were installed at the intersection points of the base with the perpendicular lines at the required distances from the first point (Fig. 2F). The primary coordinates of all landmarks are presented in Suppl. material 2.

The primary Procrustes analysis was performed with the MorphoJ program (Klingenberg 2011). Procrustes ANOVA was used to average the right and left sides of each segment. Both allometry-free analysis and isometry-free analysis (Viacava et al. 2023) were performed. However, the difference between the two methods was insignificant, so only allometry-free analysis (basic analysis in the MorphoJ program) was further used. For further analysis, data in Procrustes coordinates were exported from the saved “.morphoj” file using the Notepad++ text editor (Suppl. material 1). For further analysis, nMDS based on Euclidean’s distance in the PAST4 program was used. The coordinates of the outlines of P4 Exp3 and P4 Enp3 for MorphoJ were obtained from one of the photographs in ImageJ (Abràmoff et al. 2004) using a short macro (Roi.getSplineAnchors(xx, yy); Array.print(xx); Array.print(yy);). For visual comparison of the shapes of segments of different species, outlines (wireframes for basis) of each element (two per individual) were exported from MorphoJ in “.svg” format. Then the outlines of one species were superimposed on each other in CorelDraw.

### ﻿Integumental pore pattern analysis

The cephalothorax of representatives of the genus *Eucyclops* contains more than 200 integumental organs (Alekseev et al. 2006); therefore, the study of the pattern of integumental organs requires special methods. This can be the dissolution of the internal tissues and subsequent drawing (Alekseev et al. 2006), the use of scanning electron microscopy (Baribwegure et al. 2001), or various types of staining (Fleminger 1973). Drawing without special methods is possible (Karanovic et al. 2013), but it requires a lot of time and effort.

In this study, Congo Red staining was used, the same method used for staining for the habitus photographs, and subsequent fluorescence microscopy was used. Unfortunately, the thoracic somites were not stained well enough, so only the cephalothorax was studied. Mapping of the integumentary organs was performed in the following order:

1. Staining of whole individual;
2. Separation of the cephalothorax and its transfer on a slide, under a separate cover slip;
3. Photographing the cephalothorax in nine different projections (frontally, dorsally, ventrally, fronto-laterally on both sides, laterally on both sides, dorso-laterally on both sides) with different focal lengths (with a lens shift of 3 μm)
4. In each resulting series of photographs in Adobe Photoshop, integumentary organs were marked on each layer (on average ~ 25–30 layers);
5. Homology of elements between different projections was conducted.

A total of four cephalothoraxes were studied from both sides (left and right). We attempted to establish homology between the maps from the article of Alekseev et al. (2006) and our data. However, this was only possible for the dorsal elements, which were numbered accordingly. The groups mainly consist of one pore and one sensillum. However, the difference between a pore and a sensillum in photographs is not always obvious, so in some cases this rule may not apply. For the same reason, we refrained from separating these elements in the description and used the general term “integumental organ”. Depending on which projection the organ was best seen on, four regions were distinguished for convenience: frontal (F), dorsal (D), lateral (L), and ventral (V).

For comparative analysis, a matrix of how the number of organs contained in each group, on each side of the cephalothorax, was constructed (Suppl. material 1). Unpaired groups were recorded with the same number for each side. An average matrix between the left and right sides of one individual was also constructed (Suppl. material 1). Clustering based on the Euclidean distance was used to compare the individuals.

## ﻿Results

### ﻿Molecular analysis

We obtained the COI sequences of seven individuals of *E.sibiricus* sp. nov. from the Lena River Delta and the Putorana Plateau, including four paratypes (342–583 bp). The sequences were deposited in GenBank under the NCBI accession numbers OR917924–OR917930 (Table 3). The obtained sequences, trace files of COI fragments and individual data are available in the BOLD project SIB.

**Table 3.** The NCBI accession numbers of *Eucyclopssibiricus* sp. nov. sequences.

Individual Catalog #	COI	ITSn	18S rRNA	GenSeq Nomenclature
ch5 (paratype)	OR917926	OR941791	OR918477	genseq-2 COI, ITSn, 18S rRNA
ch6 (paratype)	OR917925	–	–	genseq-2 COI
ch7 (paratype)	OR917928	–	–	genseq-2 COI
ch8 (paratype)	OR917929	–	–	genseq-2 COI
K1-4 (non-type individual voucher)	–	OR941793	OR918476	genseq-3 ITSn, 18S rRNA
ch1 (non-type individual voucher)	OR917930	–	–	genseq-3 COI
ch2 (non-type individual voucher)	OR917924	OR941789	–	genseq-3 COI, ITSn
ch3 (non-type individual voucher)	OR917927	OR941790	OR918478	genseq-3 COI, ITSn, 18S rRNA

A search of the BOLD database using COI revealed that *E.sibiricus* sp. nov. matches to an *E.speratus* unpublished sequences with a similarity range of 90.77–99.3%. The sequences most closely related to *E.sibiricus* sp. nov. from the BOLD database was that of *E.speratus* from the Irkutsk region. The *Eucyclops* sequences from the GenBank database were included in the analyses, including those of *E.speratus* from Sakhalin Island, the Irkutsk region, the Volgograd reservoir, and St. Petersburg. The mean interpopulation p-distances between *E.sibiricus* sp. nov. individuals from the Lena River Delta and the Putorana Plateau were 1.6%. The interpopulation p-distances between *E.speratus* individuals from four populations ranged from 2.8 to 14.6%. The most genetically close were two populations from the European part of Russia (St. Petersburg / the Volgograd reservoir – 2.8%) and two populations from Siberia and the Far East (the Irkutsk region / Sakhalin Island – 4.6%). The interspecific p-distances between *E.sibiricus* sp. nov. and *E.speratus* individuals from different populations ranged from 0.8 to 14.4% (Table 4). The most genetically close were *E.sibiricus* sp. nov. individuals from the Lena River Delta and the Putorana Plateau and two *E.speratus* individuals from Sakhalin Island and the Irkutsk region (PQ216005, PQ216005); their p-distances ranged from 0.8 to 5.1%. The p-distances between *E.sibiricus* sp. nov. individuals from the Lena River Delta and the Putorana Plateau and *E.speratus* individuals from St. Petersburg were 11.4–14.4%. The intraspecific model-corrected genetic distances (Tamura 3-parameter) were identical to the p-distances for *E.sibiricus* sp. nov. and *E.speratus* from St. Petersburg and the Volgograd reservoir, while being slightly greater for *E.speratus* from the Far East and the Irkutsk region (4.8%). The interspecific model-corrected distances between *E.sibiricus* sp. nov. individuals from the Lena River Delta and the Putorana Plateau and *E.speratus* from St. Petersburg and Volgograd reservoir ranged from 12.7 to 16.4%, while those between *E.sibiricus* sp. nov. individuals from the Lena River Delta and the Putorana Plateau and *E.speratus* individuals from the Irkutsk region and the Far East ranged from 0.8 to 5.3%.

**Table 4.** The p-distances (in %) between *E.speratus* and *E.sibiricus* individuals from COI sequences.

Sequences #	NCBI ID	Species	Population	Sequences #									
				1	2	3	4	5	6	7	8	9	10
1	PQ216002	*E.speratus*	St. Petersburg										
2	KC627333	*E.speratus*	St. Petersburg	2.8									
3	PQ216000	*E.speratus*	Sakhalin Island	13.5	13.2								
4	PQ216005	*E.speratus*	Irkutsk region	14.6	14.6	4.6							
5	OR917926	*E.sibiricus*	Lena River Delta	14.2	14.1	4.2	1.3						
6	OR917929	*E.sibiricus*	Lena River Delta	14.1	14.4	4.2	1.2	0.0					
7	OR917928	*E.sibiricus*	Lena River Delta	13.0	13.4	4.2	1.0	0.0	0.0				
8	OR917924	*E.sibiricus*	Putorana Plateau	12.0	13.0	4.5	3.5	2.7	2.7	2.7			
9	OR917925	*E.sibiricus*	Lena River Delta	11.4	12.1	3.9	0.8	0.0	0.0	0.0	2.7		
10	OR917927	*E.sibiricus*	Putorana Plateau	12.1	13.7	5.1	3.8	3.2	3.0	3.0	0.0	3.1	
11	OR917930	*E.sibiricus*	Putorana Plateau	11.9	13.5	4.5	3.3	2.7	2.5	2.5	0.0	2.6	0.0

**Figure 3.**
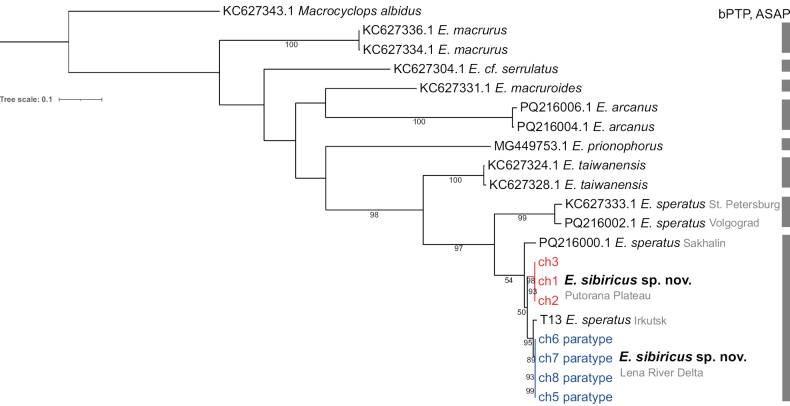
The ML phylogenetic tree is based on the COI mtDNA gene. The number in the node is the bootstrap support value. The colored numbers indicate the sampling locations. Sequences from GenBank are preceded by their access numbers. The bars adjacent to the tree indicate the species delimited by ASAP and bPTP methods.

On the COI phylogenetic tree, individuals of *E.sibiricus* sp. nov. cluster with *E.speratus* from the Irkutsk region (Fig. 3). The individuals of *E.sibiricus* sp. nov. from the Putorana Plateau population are genetically distinct from those of the Lena Delta and Irkutsk region. *Eucyclopssibiricus* sp. nov. and *E.speratus* from Siberia and the Far East are sister taxa to *E.speratus* from the European Russia populations.

The ASAP and bPTP delimitation methods were applied to the COI data for the *Eucyclops* data set and yielded eight species (Fig. 3). These results are in accordance with the COI tree topology. *Eucyclopssibiricus* sp. nov. and *E.speratus* from the Irkutsk region belong to a single species, which is distinct from *E.speratus* from the European Russia. It is likely that the individuals from Sakhalin Island belong to the same species, but there is insufficient evidence.

**Figure 4.**
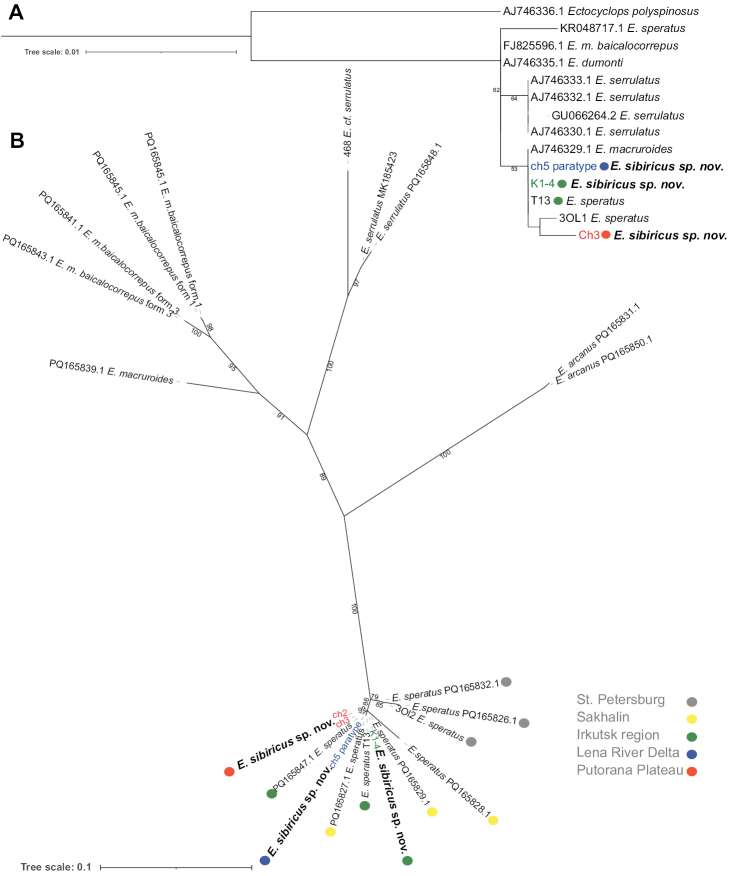
The ML phylogenetic trees are based on sequences **A** 18S rRNA (K2P+I) **B** ITSn rDNA (TNe+G4). The number in the node is the bootstrap support value. The colored circles indicate the sampling locations. Sequences from GenBank are preceded by their access numbers.

The ITSn sequences, comprising the first and second transcribed spacers and 5.8S rRNA rDNA, were determined for four individuals of *E.sibiricus* sp. nov. from the Lena River Delta, the Irkutsk region, and the Putorana Plateau populations, including one paratype. Additionally, ITSn was sequenced from one individual of *E.speratus* from St. Petersburg and individual T13 (OR941794), which were most closely related to *E.sibiricus* sp. nov. based on the COI analysis. The ITSn sequences (278–846 bp) were deposited in the GenBank database with the following accession numbers: OR941789–OR941791, OR941793 for *E.sibiricus* sp. nov., and PQ316111 for *E.speratus*. The most closely related sequences to those of *E.sibiricus* sp. nov. ITSn were found in the GenBank database through BLAST analysis. The sequences were those of *E.speratus* populations from Sakhalin Island, the Irkutsk region, and the vicinity of St. Petersburg. The mean interpopulation p-distances between individuals of *E.sibiricus* sp. nov. from the Putorana Plateau, the Lena River Delta, and the Irkutsk region were 0%. The mean interpopulation p-distances between *E.speratus* individuals from the Irkutsk region, Sakhalin and St. Petersburg populations ranged from 1.5 to 2.3%. The p-distances between *E.sibiricus* sp. nov. individuals from the Putorana Plateau, the Lena River Delta, the Irkutsk region, and *E.speratus* individuals from St. Petersburg ranged from 0.7 to 2.9% (Table 5). The p-distances between *E.sibiricus* sp. nov. individuals from the Putorana Plateau, the Lena River Delta, the Irkutsk region and *E.speratus* individuals from the Far East and the Irkutsk region ranged from 0 to 6.2%. Within groups, the model-corrected genetic distances (JC) were identical to the p-distances; between groups, such values were observed to be similar. The model-corrected distances between *E.sibiricus* sp. nov. individuals from the Putorana Plateau, the Lena River Delta, the Irkutsk region, and *E.speratus* individuals from St. Petersburg ranged from 0.7 to 2.9%. The p-distances between *E.sibiricus* sp. nov. individuals from the Putorana Plateau, the Lena River Delta, and the Irkutsk region and *E.speratus* individuals from the Far East and the Irkutsk region ranged from 0 to 6.5%. The entire length of ITS1 of three individuals of *E.sibiricus* sp. nov. from the Putorana Plateau and Irkutsk region populations (Ch2, Ch3, K1-4) is 391 bp, while in one sequence of *E.speratus* from St. Petersburg it is 388 bp. The entire length of ITS2 of two individuals of *E.sibiricus* sp. nov. from the Putorana Plateau population (Ch2, Ch3) was 274 bp. The entire length of ITS2 for *E.speratus* is not completely determined. The ITSn phylogenetic tree is consistent with the COI tree and indicates that *E.sibiricus* sp. nov. forms a monophyletic group with *E.speratus* from the Far East and the Irkutsk region, which includes one of the most divergent sequences of *E.speratus* from the Far East (PQ165828.1; Fig. 4B). This group is genetically closely related to the population of *E.speratus* from St. Petersburg. *Eucyclopssibiricus* and *E.speratus* are sister taxa to *E.arcanus* Alekseev, 1990.

**Table 5.** The p-distances (in %) between *E.speratus* and *E.sibiricus* individuals from ITSn sequences.

Sequences #	NCBI ID	Species	Population	Sequences #										
				1	2	3	4	5	6	7	8	9	10	11
1	PQ165832	*E.speratus*	St. Petersburg											
2	PQ316111	*E.speratus*	St. Petersburg	0.0										
3	PQ165826	*E.speratus*	St. Petersburg	1.0	0.8									
4	PQ165827	*E.speratus*	Sakhalin	0.8	1.1	2.3								
5	PQ165828	*E.speratus*	Sakhalin	3.5	5.7	3.7	2.8							
6	PQ165829	*E.speratus*	Sakhalin	0.7	1.1	2.0	0.3	2.5						
7	PQ165847	*E.speratus*	Irkutsk region	0.4	0.0	1.7	0.6	0.9	0.2					
8	OR941794	*E.speratus*	Irkutsk region	1.6	2.9	2.4	0.0	8.1	0.0	NA				
9	OR941793	*E.sibiricus*	Irkutsk region	0.9	2.0	2.4	0.2	3.4	0.0	0.0	0.0			
10	OR941789	*E.sibiricus*	Putorana Plateau	0.8	2.2	2.1	0.4	2.7	0.1	0.2	0.4	0.2		
11	OR941790	*E.sibiricus*	Putorana Plateau	0.7	2.0	2.0	0.3	2.6	0.0	0.2	0.0	0.0	0.0	
12	OR941791	*E.sibiricus*	Lena River Delta	1.8	2.5	2.9	0.0	6.2	0.0	0.0	0.0	0.0	0.0	0.0

NA – the sequences do not overlap with one another.

We obtained the sequences of the 18S gene for four specimens of *E.sibiricus* sp. nov. (one paratype from the Lena River Delta, two specimens from the Irkutsk region, one specimen from the Putorana Plateau, 406–550 bp), and one specimen of *E.speratus* (549 bp) from St. Petersburg. The sequences were deposited in the GenBank database and assigned the following numbers OR918475–OR918478 for *E.sibiricus* sp. nov. and OR918479 for *E.speratus*. The intraspecific p-distances among *E.sibiricus* sp. nov. from the Putorana Plateau, the Irkutsk region, and the Lena River Delta were 0%, while those among *E.speratus* from Korea, St. Petersburg, and the Irkutsk region were 0.5%. The highest genetic diversity between *E.speratus* individuals is represented by one individual from St. Petersburg and one from Korea. The mean p-distances between *Eucyclops* species from 18S varied from 0% to 0.4%. The mean p-distances between *E.speratus* from Korea, St. Petersburg, and the Irkutsk region and *E.sibiricus* sp. nov. from the Putorana Plateau, the Irkutsk region, and the Lena River Delta are 0.3%. The model-corrected genetic distances (JC+G) within and between species are consistent with the p-distances. On the 18S tree, *E.sibiricus* sp. nov. forms a cluster with *E.speratus* from St. Petersburg and *E.macruroides* (Lilljeborg 1901) from St. Petersburg, but with low bootstrap support due to the low evolutionary rate of the fragment.

### ﻿Morphological analysis

Comparison of qualitative morphological characters did not yield any results. Both species have identical sets of spinules and setules groups on the most taxonomically important parts: coxobasis of the antenna, maxillular palp, coxa, and intercoxal sclerite P4.

**Figure 5.**
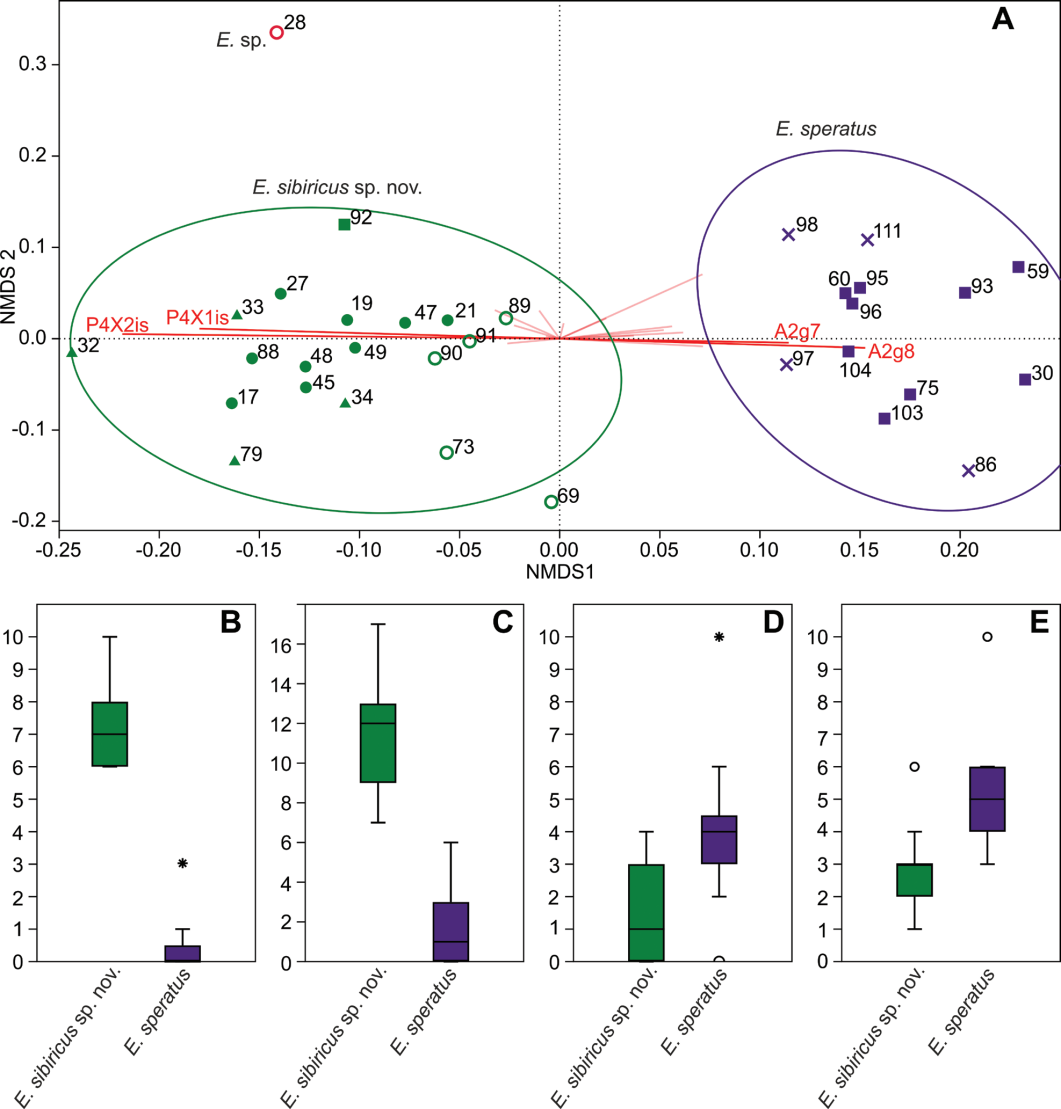
Comparison of quantitative morphological characters **A**nMDS of quantitative morphological characters, stress 0.1825. Gower index. Concentration ellipses 90% **B**–**E** boxplots for the most important characters **B** number of setules on the inner side of P4 Exp2 (P4X2is) **C** number of setules on the inner side of P4 Exp1 (P4X2is) **D** number of spinules of group 8 of the coxobasis of the antenna (A2g8) **E** number of spinules of group 7 of the coxobasis of the antenna (A2g7). Symbols: empty circle – North/Central Yakutia; filled dots – Lena River Delta; triangles – Anabar/Putorana Plateau; squares – Udmurtia/Tatarstan; crosses – Saint Petersburg.

The quantitative analysis of morphological characters used as a basis the number of spinules/setules in each group. In the nMDS graph, we see a fairly clear division of all studied individuals into two groups: *E.sibiricus* sp. nov. and *E.speratus* (Fig. 5A). One individual (N°28) in the graph is located far from the others, so we cannot reliably assign it to any species. Individuals from the same sample (69, 73) are morphologically closer to *E.sibiricus* sp. nov. It can be assumed that individual N°28 is either a separate species or a malformed individual of *E.sibiricus* sp. nov.

When plotting the characters vectors on the nMDS plot, we can see that only four characters have vectors parallel to the differences between species. In order of decreasing importance, these are:

1. Number of setules on the inner side of P4 Exp2 (P4X2is) (Fig. 5B);
2. Number of setules on the inner side of P4 Exp1 (P4X2is) (Fig. 5C);
3. Number of spinules of group 8 of the coxobasis of the antenna (A2g8) (Fig. 5D);
4. Number of spinules of group 7 of the coxobasis of the antenna (A2g7) (Fig. 5E).

At the same time, the features concerning the antenna largely overlap in different species. However, the number of setules on the inner side of P4 Exp1–Exp2 does not overlap. The total number of setules on these two segments is probably the most reliable feature distinguishing the two species.

In general, the individuals were well separated geographically: almost all individuals from Asia were assigned to *E.sibiricus* sp. nov., whilst almost all from Europe were assigned to *E.speratus* (Fig. 5A). Only one individual (i.e., N° 92) from Tatarstan was close to the Asian individuals. Moreover, it was found in the same sample as *E.speratus* individuals N° 75, 103, 104. Unfortunately, no other individuals similar to N° 92 were found. Cohabitation, discrete morphological features, and the absence of intermediate forms confirm that these two groups are different species with a partially overlapping range.

### ﻿Linear morphometrics

**Figure 6.**
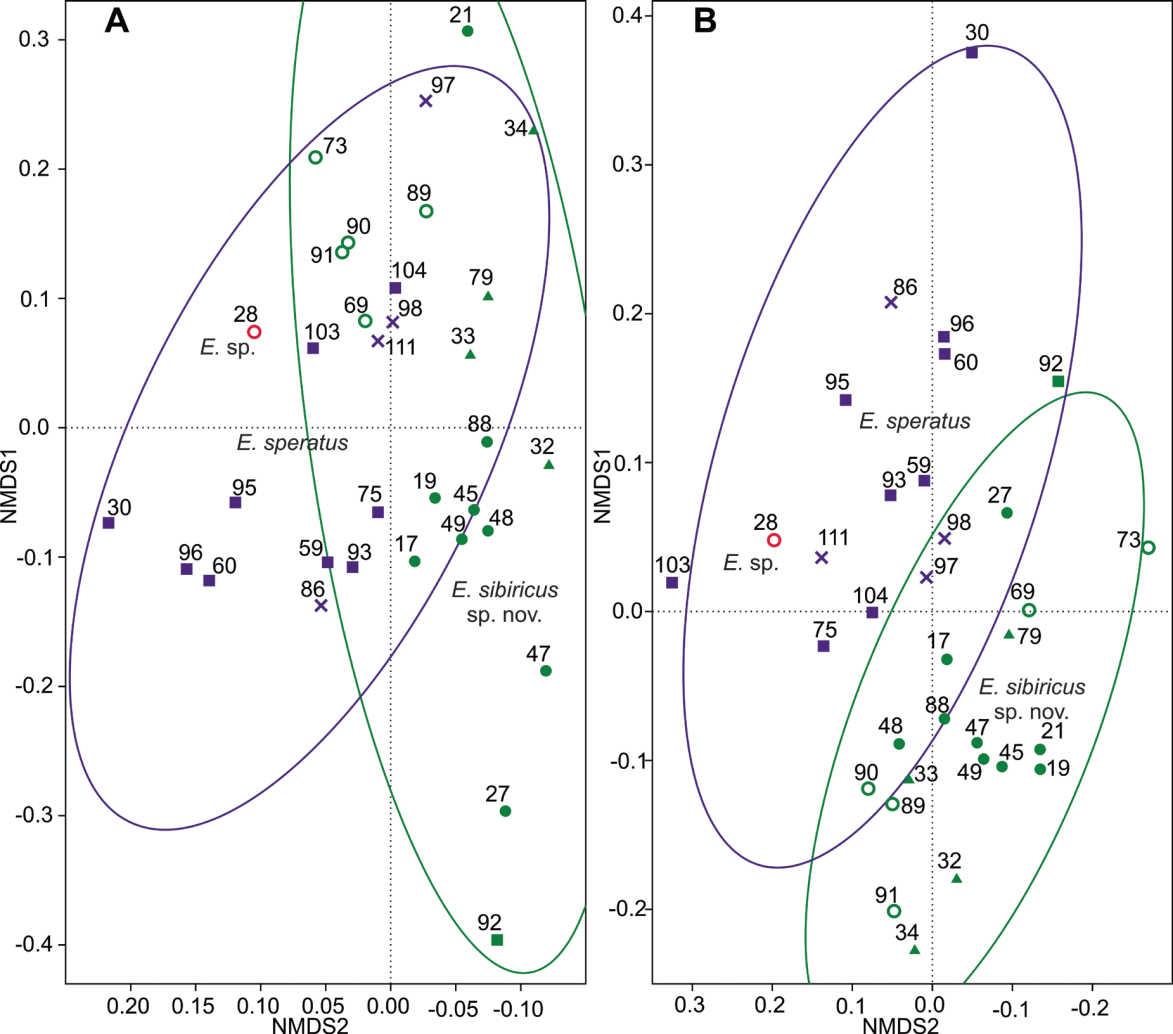
Linear morphometrics **A**nMDS based on absolute values, stress 0.1081 **B**nMDS based on ratios of measurements, stress 0.2228. Gower index. Concentration ellipses 90%. Symbols: empty circle – North/Central Yakutia; filled dots – Lena River Delta; triangles – Anabar/Putorana Plateau; squares – Udmurtia/Tatarstan; crosses – Saint Petersburg.

Linear morphometry based on absolute values does not show any significant differences between the species (Fig. 6A). Linear morphometry based on the relative values (ratios between measurements) separates the two species much better (Fig. 6B). Concentration ellipses intersect only partially. Comparison by Mann-Whitney test shows that xas/x and no/n ratios differ significantly between species (Table 6). However, their ranges of variation overlap, so these characters can hardly be used for identification, but only as an additional discriminant character. In particular, the outer seta P4 Enp3 in *E.sibiricus* sp. nov. reaches approximately halfway the length of the outer spine, while in *E.speratus* it reaches almost to the end of this spine.

**Table 6.** Ratios of measurements in *E.sibiricus* sp. nov. and *E.speratus* (for the explanation of the abbreviations, see methods).

Ratio	*E.sibiricus* sp. nov.		*E.speratus*		Mann-Whitney p-value
	median	range	median	range	
xa/x	0.92	0.80–0.99	0.95	0.82–1.07	0.11569
xas/x	1.11	1.02–1.22	1.23	1.14–1.39	0.00003
xas/xa	1.22	1.10–1.38	1.30	1.14–1.46	0.03827
n/ns	2.78	2.29–3.03	2.53	2.29–2.71	0.00573
no/n	0.63	0.57–0.77	0.76	0.68–0.83	0.00001
nai/nao	1.51	1.40–1.66	1.47	1.38–1.51	0.00573
nai/n	1.05	0.94–1.09	1.01	0.89–1.13	0.28267
cr/cs	5.91	4.76–7.12	6.04	5.40–6.33	0.23426
crr/cr	0.16	0.08–0.30	0.20	0.10–0.36	0.00573
cri/cro	1.26	1.12–1.51	1.18	1.07–1.28	0.02194
cri/cr	0.55	0.49–0.61	0.54	0.47–0.58	0.32037

### ﻿Geometric morphometrics

**Figure 7.**
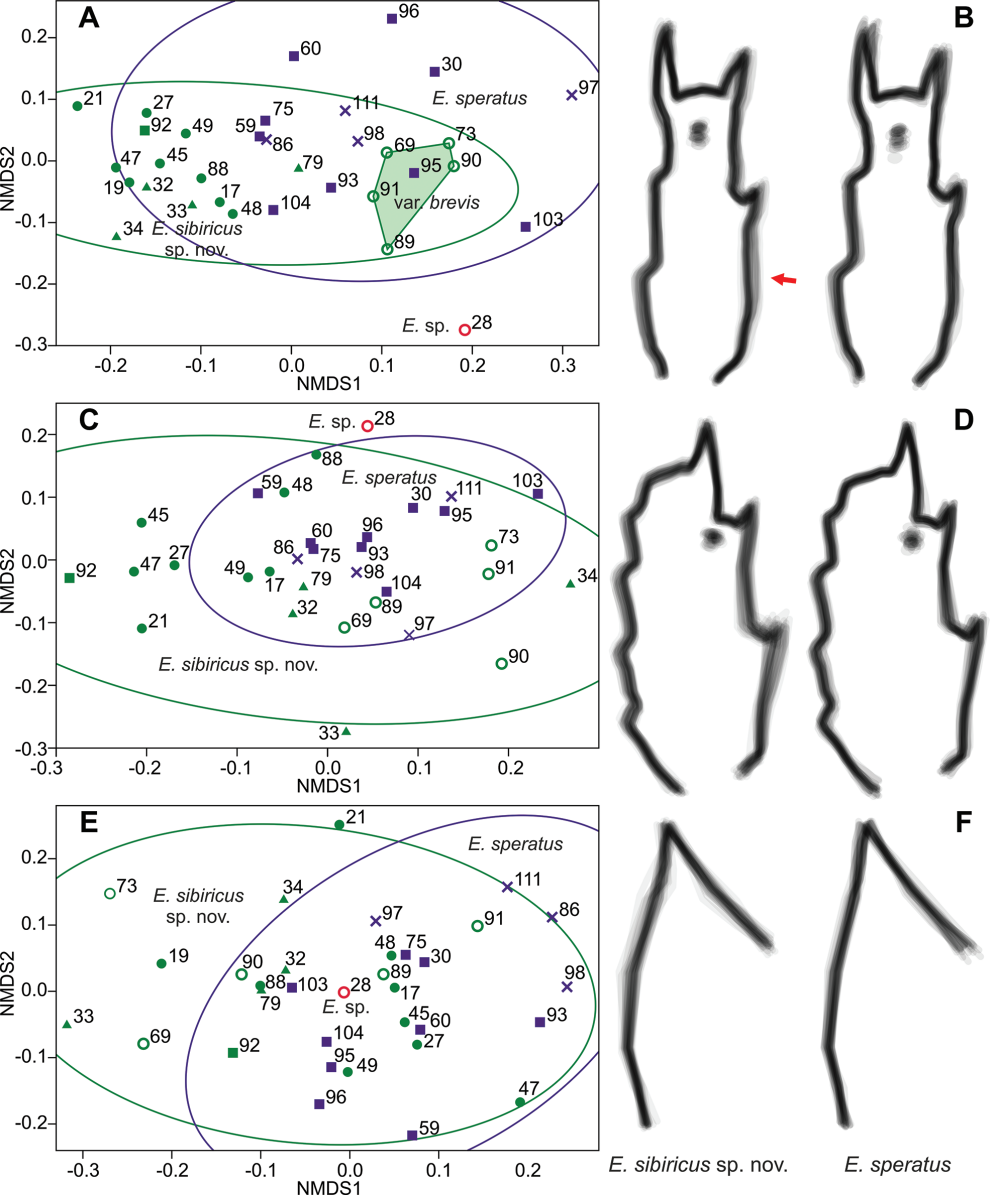
Geometric morphometrics **A, C, E**nMDS based on Procrustes coordinates of landmarks. Euclidean index. Concentration ellipses 90%. **B, D, E** shape comparisons of superimposed outlines of all individuals of the two species *E.sibiricus* sp. nov. and *E.speratus*. **A**nMDS based on P4 Enp3, stress 0.1804 **B** shape of P4 Enp3 **C**nMDS based on P4 Exp3, stress 0.1555 **D** shape of P4 Exp3 **E**nMDS based on the P4 inner side of basis, stress 0.1804 **F** P4 inner side of basis. Symbols: empty circle – North/Central Yakutia; filled dots – Lena River Delta; triangles – Anabar/Putorana Plateau; squares – Udmurtia/Tatarstan; crosses – Saint Petersburg.

Geometric morphometrics in general does not allow us to distinguish between *E.sibiricus* sp. nov. and *E.speratus* (Fig. 7). The greatest differences are recorded for the P4 Enp3 (Fig. 7A); most of the *E.sibiricus* sp. nov. individuals are concentrated in the left part of the plot and most of the *E.speratus* individuals are in the right part. If we compare the average shapes of the segments, we can see that *E.speratus* has a wider segment with a variable direction of the apical spiniform processes (Fig. 7B). *Eucyclopssibiricus* has an elongated segment with the most variable proximal part (arrowed in Fig. 7B).

Several individuals of *E.sibiricus* sp. nov. (i.e., individuals N° 69, 73, 89, 90, 91) differ significantly from other individuals of this species and are marked in Fig. 7A. All of them were found in two samples in northern and central Yakutia. It should be noted that according to other analyses (quantitative morphological, linear morphometrics), these individuals do not differ from other *E.sibiricus* sp. nov. Therefore, we distinguish it as a variety: E.sibiricussp. nov.var.brevis, which does not have a taxonomic status. Unfortunately, we do not have the genetic data on the individuals from these populations.

For the geometric morphometrics of P4 Exp3, the measurements of this feature are highly scattered in *E.sibiricus* sp. nov. (Fig. 7C). In the nMDS plot of this feature, not only the individuals of var. brevis are shifted towards *E.speratus*, but also the individuals of *E.sibiricus* sp. nov. from the Putorana Plateau and the Anabar Plateau (triangles on figures). The available genetic data show that such variability is intraspecific. The average forms of the segment show the blurred lines of the form of *E.sibiricus* sp. nov., which indicates high intraspecific variability (Fig. 7D). On average, *E.speratus* has a shorter and wider segment than *E.sibiricus* sp. nov.

The geometric morphometry of the inner side of the basis of P4 (Fig. 7E) was the least differing morphometric character, and showed no discernible pattern. The average forms only show a slightly more convex middle part in *E.sibiricus* sp. nov. (Fig. 7F).

### ﻿Integumental organs pattern of cephalothorax

**Figure 8.**
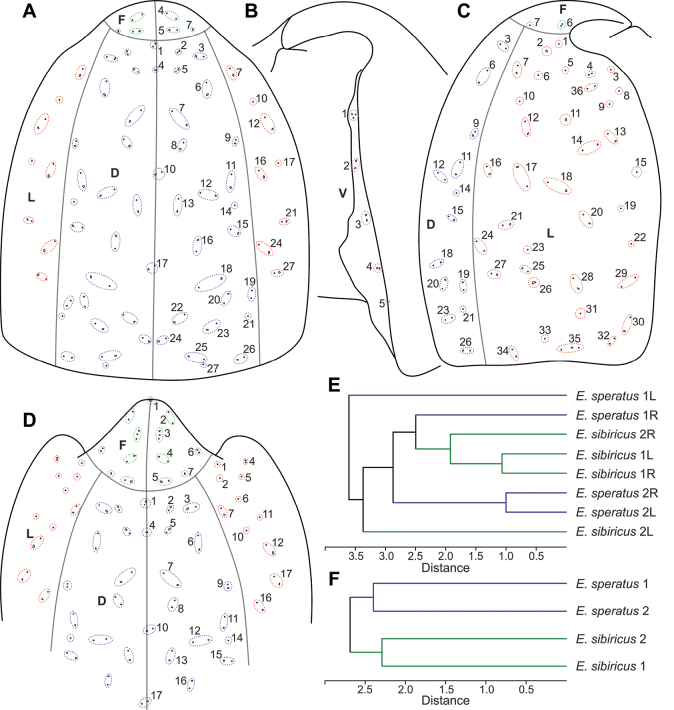
Integumental organs pattern analysis **A–D** general pattern of arrangement of integumental organs of the cephalothorax in *E.sibiricus* sp. nov. and *E.speratus***A** dorsal side **B** ventral side **C** lateral side **D** frontal side **E** dendrogram of similarity between different sides of the cephalothorax of the studied individuals. Euclidean index **F** dendrogram of similarity between average cephalothoraxes. Euclidean index

In the general scheme of the arrangement of integumental organs, only those elements that were recorded more than once are marked, since additional organs recorded only once could be deviations or photographic artifacts. A total of 254 pores and sensilla were recorded and are marked in Fig. 8A–D. The dorsal region includes 27 groups with 42 paired and seven unpaired organs (Fig. 8A). The ventral region includes five groups with ten paired organs (Fig. 8B). The lateral region includes 36 groups with 56 paired organs (Fig. 8C). The frontal region includes seven groups with 12 paired and one unpaired organ (Fig. 8D).

Each pore and sensillum can be considered a separate feature. If we compare the right and left sides of the studied cephalothoraxes separately, the species do not group together due to too high variability (Fig. 8E). However, the data averaged from all the individuals allows separating the species (Fig. 8F): the distance between individuals of the same species is significantly higher than the distance between two different species. The only group of pores that differs in different species in the available sample, is group L36. It is always absent in *E.sibiricus* sp. nov. and is present in *E.speratus* on three of four sides.

### ﻿Taxonomic account

**Subclass Copepoda H. Milne Edwards, 1840**

**Order Cyclopoida Burmeister, 1834**

**Family Cyclopidae Rafinesque, 1815**

**Genus *Eucyclops* Claus, 1893**

#### ﻿ Eucyclops sibiricus sp. nov.

Taxon classificationAnimaliaCyclopoidaCyclopidae

275E12F4-54C2-57C1-9EA6-C92C85F31A14

https://zoobank.org/F7026136-6AD2-4B74-9D83-CC377E39548D

Figs 9 , 10 , 11 , 12 , 13 , 14 , 15 , 16 , 17 , 18

Eucyclops speratus : Ishida and Hiruta 1999 (part): 88, figs 1, 2a–e; Lee et al. 2005: 146, fig. 5; Chertoprud et al. 2022: 6, table A1.
Eucyclops sp. nov.: Chertoprud et al. 2024: 1014, supplemental material 1: table S1.

##### Etymology.

The species was first discovered and has its main range in Siberia. The word *sibiricus* is an adjective in the masculine gender.

##### Type material.

***Holotype***: Russia • ♀ dissected on two slides; North Yakutia, Lena River Delta, Floodplain puddle; 72.38755°N, 126.48205°E; 8 Sep. 2020; E. Abramova leg; EMKSU VH 2000/1, VH 2000/2 (N°45). ***Paratypes***: Russia • ♂ dissected on one slide; North Yakutia, Lena River Delta, Floodplain puddle; 72.36874°N, 126.47595°E; 20 Aug. 2019; A. Novikov leg; EMKSU VH 2000/3. • ♀ dissected on one slide; collection data as for holotype; EMKSU VH 2000/4 (N° 47); • ♀ dissected on one slide; collection data as for holotype; EMKSU VH 2000/5 (N° 48); • ♀ dissected on one slide; collection data as for holotype; EMKSU VH 2000/6 (N° 49); • 3 ♀ and 2 ♂ preserved in 4% formalin; North Yakutia, Lena River Delta, Floodplain puddle; 72.36874°N, 126.47595°E; 20 Aug. 2019; A. Novikov leg; EMKSU VH 2000/13; • 2 ♀ dissected on one slides; collection data as for preceding; EMKSU VH 2000/7; • 1 ♂ dissected on one slide; collection data as for preceding; EMKSU VH 2000/8; • ♀ dissected on one slide; collection data as for preceding; EMKSU VH 2000/9 (N° 27); • ♀ dissected on one slides; collection data as for preceding; EMKSU VH 2000/10 (N° 88); • ♀ preserved in 96% ethanol; North Yakutia, Lena River Delta, Floodplain puddle; 72.38755°N, 126.48205°E; 18 July 2021; A. Novikov leg; GenBank: OR917926, OR941791, OR918477; EMKSU VH 2000/14 (N° ch5); • ♀ preserved in 96% ethanol; collection data as for preceding; GenBank: OR917925; EMKSU VH 2000/15 (N° ch6); • ♀ preserved in 96% ethanol; collection data as for preceding; GenBank: OR917928; EMKSU VH 2000/16 (N° ch7); • ♀ preserved in 96% ethanol; collection data as for preceding; GenBank: OR917929; EMKSU VH 2000/17 (N° ch8);• ♀ dissected on one slides; North Yakutia, Lena River Delta, Floodplain puddle; 72.38755°N, 126.48205°E; 18 July 2021; A. Novikov leg; EMKSU VH 2000/11 (N° 17); • ♀ dissected on two slides; collection data as for preceding; EMKSU VH 2000/12 (N° 19);

##### Additional material.

Russia • ♀ dissected on one slides; North Yakutia, Lena River Delta, small river; 72.11877°N, 126.98782°E; 22 July 2021; A. Novikov leg; in collection of A. Novikov (N° 21); • 3 ♀ dissected on three slides; North Yakutia, lake; 69.97000°N, 125.60158°E; 28 June 2019; A. Novikov leg; in collection of A. Novikov (N° 89, 90, 91); • 2 ♀ dissected on two slides; Central Yakutia, temporary puddles; 64.94187°N, 124.84179°E; 22 June 2019; A. Novikov leg; in collection of A. Novikov (N° 69, 73); • ♀ dissected on one slides; Anabar Plateau, small lake; 70.64073°N, 105.90506°E; 25 July 2023; A. Novikov and E. Chertoprud leg; in collection of A. Novikov (N° 79); • ♀ dissected on one slides; Putorana Plateau, swampy lake; 68.60415°N, 94.05641°E; 7 Aug. 2021; E. Chertoprud leg; in collection of A. Novikov (N° 32); • ♀ preserved in 96% ethanol; Putorana Plateau, swampy lake; 68.60415°N, 94.05641°E; 7 Aug. 2021; E. Chertoprud leg; GenBank: OR917930; EMKSU VH 2000/18–20 (N° ch1); • ♀ preserved in 96% ethanol; collection data as for preceding; GenBank: OR917924, OR941789; EMKSU VH 2000/18–20 (N° ch2); • ♀ preserved in 96% ethanol; collection data as for preceding; GenBank: OR917927, OR941790, OR918478; EMKSU VH 2000/18–20 (N° ch3); • ♀ dissected on one slides; Putorana Plateau, swampy lake; 68.60344°N, 94.05875°E; 7 Aug. 2021; E. Chertoprud leg; in collection of A. Novikov (N° 33); • ♀ dissected on one slides; Putorana Plateau, swampy lake; 68.60029°N, 94.05300°E; 7 Aug. 2021; E. Chertoprud leg; in collection of A. Novikov (N° 34); • ♀ dissected on one slides; Tatarstan republic, Naberezhnie Morkvashi village, puddle on stream; 55.76324°N, 48.85700°E; 28 June 2020; A. Novikov and D. Sharafutdinova leg; in collection of A. Novikov (N° 92); • ♀ on the electron microscope stage no. 90134; Irkutsk region, Tulun district, Aleksandrovka village, river; 55.3905°N, 100.8231°E; 25 July 2021; T. Mayor leg; LI SB RAS T13); • ♀ preserved in 96% ethanol; Irkutsk region, Kuitun District, pond; 54.34722°N, 101.52861°E; June 2021; G. Dzyba leg; GenBank: OR941793, OR918476; in collection of T. Mayor (N° K1-4)

##### Diagnosis.

Proctodeum with rows of setules. Caudal ramus with serra and dorsal pore. Length/width ratio of caudal rami 5.2–7.0. Ratio of lengths of caudal seta VI to seta III 1.07–1.47. Antennule 12-segmented, with hyaline membrane on three distal segments. Hyaline membrane finely serrated. Antennal coxobasis with groups of spinules 3, 4, 5, 6, 7, 8, 9, 10, 11, 12, 14, 15, 16. Maxillular basis with group of spinules. P1–P4 Exp1 without spinules/setules on outer side. P1–P4 Exp2 with setules on outer side. P4 Exp1 with 8–17 inner setules. P4 Exp2 with 6–10 inner setules.

##### Description.

**Figure 9.**
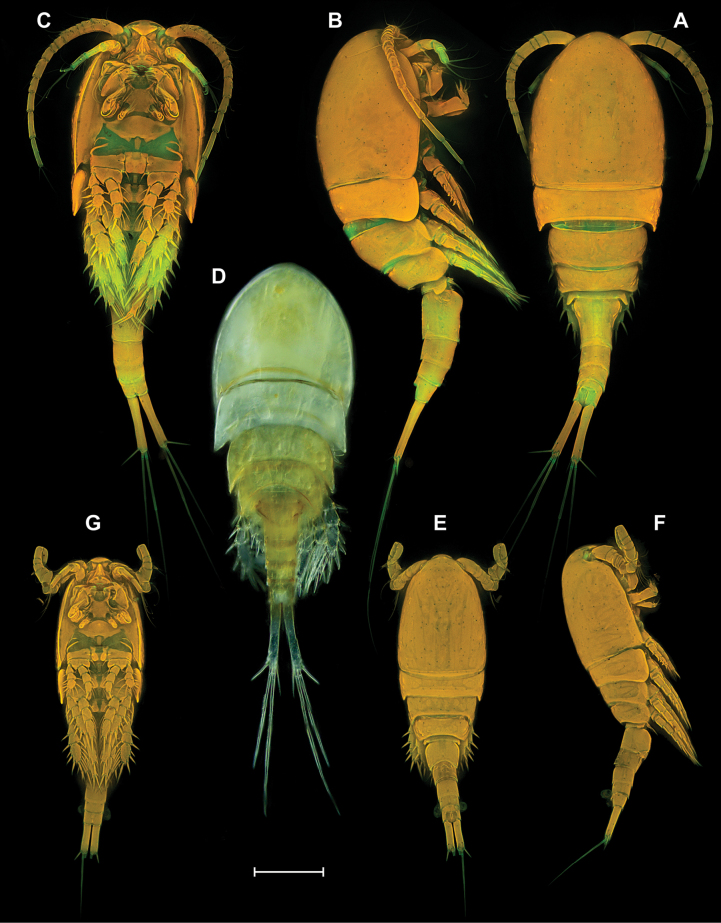
*Eucyclopssibiricus* sp. nov. Female, habitus **A** paratype, dorsal **B** paratype, lateral **C** paratype, ventral **D** intravital coloration. Male paratype, habitus: **E** dorsal **F** lateral **G** ventral. Scale bar: 200 μm.

**Female. *Body elongated*** (Fig. 9A–C). Total body length from anterior margin of rostrum to posterior margin of caudal rami: mean 1279 µm (*n* = 3). Cephalothorax wider than first and second free somites, largest width 408 µm (*n* = 1), surface with 251 pores and sensilla, with all groups, except group 36 (Fig. 8A–D). Naupliar eye present, color red or orange (Fig. 9D). Rostrum fused with cephalothorax. Posterior margin of cephalothorax and all pedigerous somites smooth. Body color yellowish, except second pedigerous somite, this somite white (Fig. 9D).

**Figure 10.**
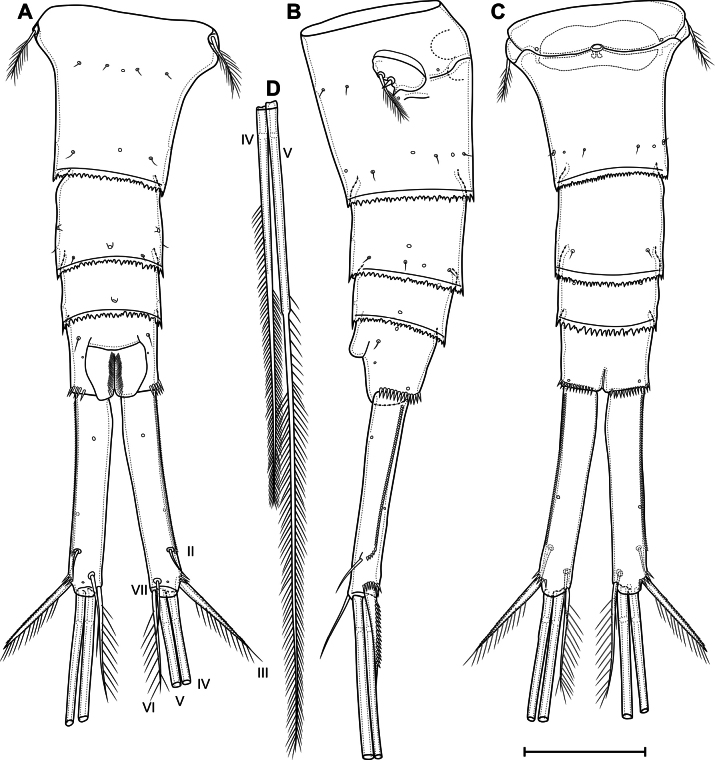
*Eucyclopssibiricus* sp. nov. Female, holotype: **A** abdomen, dorsal **B** abdomen lateral **C** abdomen ventral **D** apical caudal setae, dorsal. Scale bar: 100 μm.

**Figure 11.**
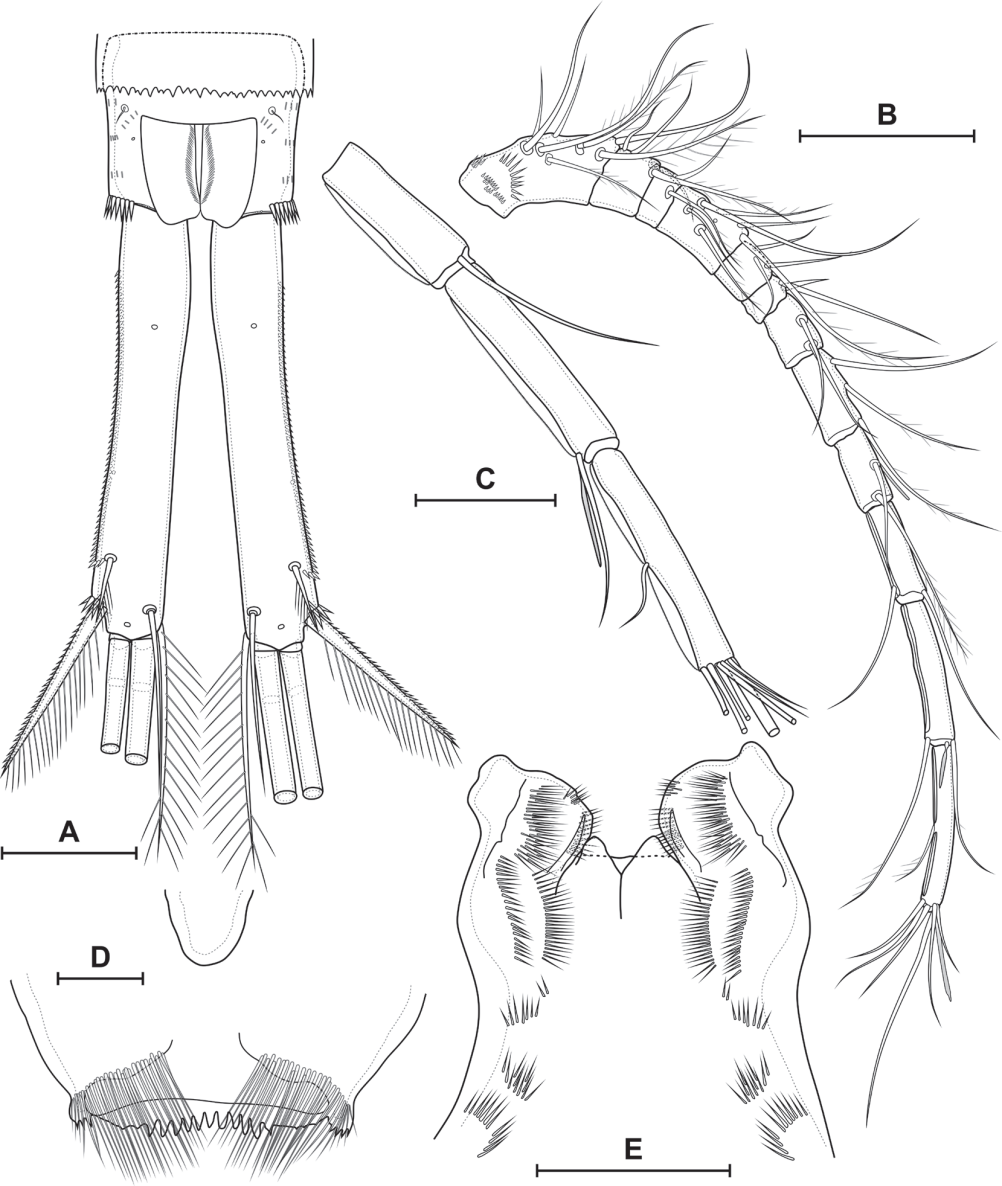
*Eucyclopssibiricus* sp. nov. Female var. **A**brevis, caudal rami, dorsal **B** holotype, antennule **C** holotype, distal segments of antennule **D** holotype, labrum, anterior **E** holotype, paragnaths, posterior. Scale bars: 100 μm (**B**); 50 μm (**A, C, E**); 20 μm.(**D**).

***Abdomen*** (Figs 10A–C, 11A) consisting of genital double-somite, two free abdominal somites, and anal somite with caudal rami. Genital, second, and third abdominal somites on posterior margin serrated. Genital double-somite consists of last thoracic somite and first abdominal somite; longer than wide; anterior part with two dorsal pairs of sensilla, dorsal unpaired pore, lateral and ventral pairs of pores; posterior part with three pairs of sensilla, two pairs of pores and dorsal unpaired pore.

***P6*** (Fig. 10A, B) lateral, formed plate, partially fused with somite; with one long plumose seta, one short spine and small spiniform process. Genital field (Fig. 10C) wide, bean-shaped. Copulatory pore medial, located on notable suture cord. Copulatory duct short, chitinised. Anterior and posterior chambers of seminal receptacle equal in wide, narrow. Two egg sacks attached to gonopore area.

***Second abdominal somite*** (Fig. 10A–C) with three pairs of sensilla, two pairs of pores, and dorsal unpaired pore. Third abdominal somite (Fig. 10A–C) with dorsal unpaired pore, pairs of lateral and ventral pores. Anal somite with one dorsal pair of sensilla: one dorsal, one anterolateral, one lateral, and one ventral pairs of pores; with or without small posterior spinules near sides of proctodeum; with ventro-lateral posterior rows of spinules; proctodeum with rows of setules. Anal operculum slightly protruding.

***Caudal rami*** (Figs 10A–C, 11A). Length/width ratio 6,5 in holotype (5.2–7.0, median 5.91, *n* = 19). Ramus with one ventral and two dorsal pores; with longitudinal row of small spinules (serra); with spinules at base of seta III. Serra extending from base of seta II to anterior 8% of ramus in holotype (8%–30%, median 16%, *n* = 19). Seta I absent. Seta II dorsolateral, small. Seta III spiniform, with spinules on outer side and long setules on inner side. Apical setae IV and V (Fig. 10D) long; bipinnate; with fracture planes; proximal part bare, medial part with rare long spinules, distal part with dense long spinules; length ratios 1 and 1.63, respectively. Seta VI with several inner setules and with or without outer setules; longer than seta III (ratio of lengths of seta VI to seta III 1.07–1.47, median 1.26, *n* = 19). Seta VII biarticulate.

***Antennule*** (Fig. 11B, C) 12-segmented; extending to second pedigerous somite. Armature formula as follows: 1-[8],2-[4],3-[2],4-[6],5-[4],6-[2],7-[2],8-[3],9-[2+ae],10-[2],11-[2+ae],12-[7+ae]. Segment 1 with four rows of spinules. Segment 4 with one pore. Three distal segments with finely serrate hyaline membrane (Fig. 11C); proximal part of membrane of distal segment with 90 small denticles. Aesthetascs located on segments 9, 11, and 12. One seta on segment 6 spiniform. One apical seta on segment 12 fused basally to aesthetasc.

**Figure 12.**
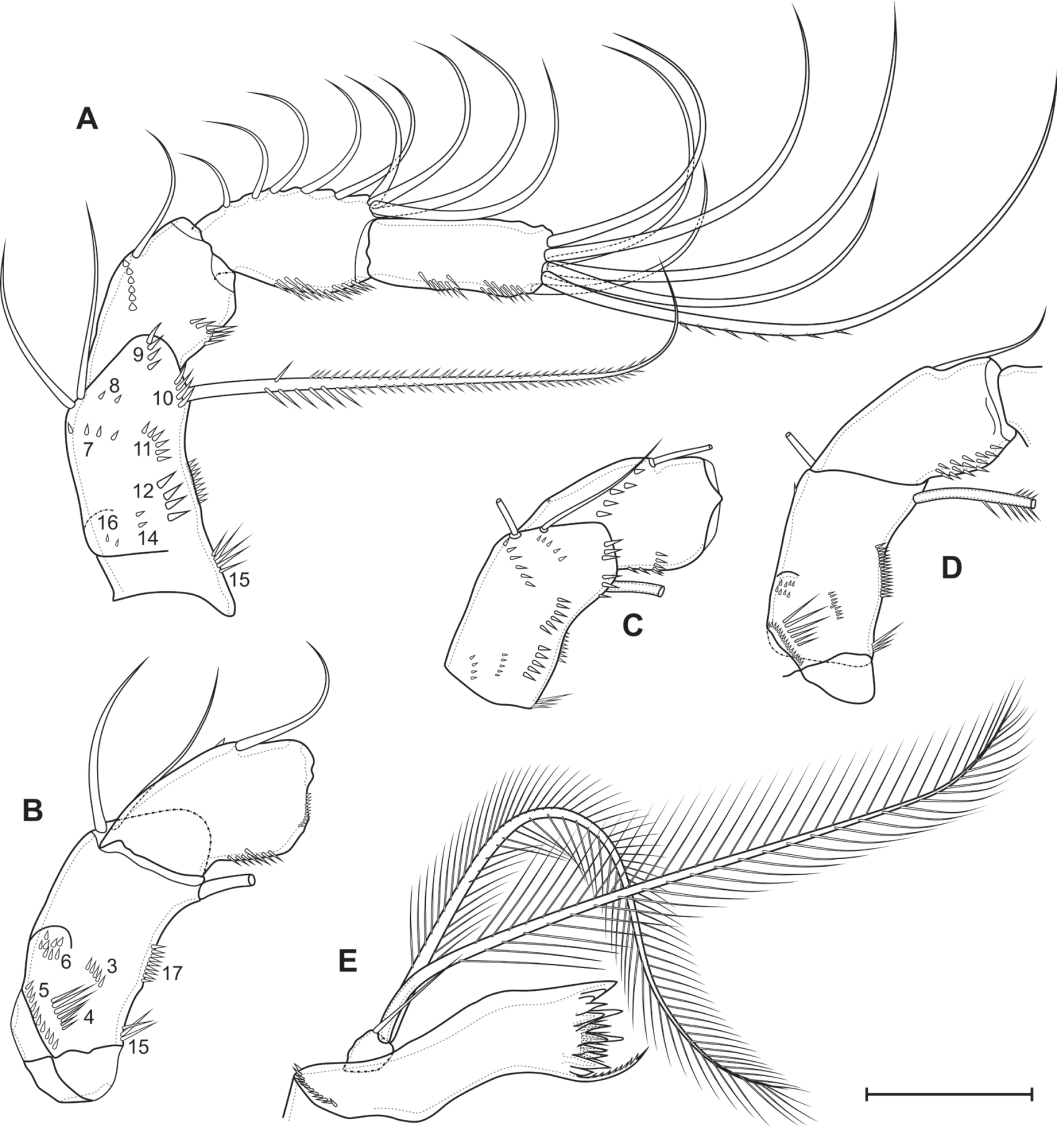
*Eucyclopssibiricus* sp. nov. Female **A** holotype, antenna, anterior **B** holotype, coxobasis and first endopodal segment of antenna, posterior **C** var. brevis, coxobasis and first endopodal segment of antenna, anterior **D** var. brevis, coxobasis and first endopodal segment of antenna, posterior **E** mandible. Scale bar: 50 μm.

***Antenna*** (Fig. 12A–D) four-segmented include coxobasis and three-segmented endopod. Coxobasis rectangular; with three setae, one of these exopodal long pinnate seta; with groups of spinules 3, 4, 5, 6, 7, 8, 9, 10, 11, 12, 14, 15, 16. Number of spinules of group 7: 1–6 (median 3, *n* = 19). Number of spinules of group 8: 0–5 (median 1, *n* = 19). First endopodal segment with one naked seta; one postero-lateral and postero-medial rows of spinules. Second endopodal segment with nine naked setae on inner margin, these setae longer from proximal to distal; with row of spinules along outer margin. Third endopodal segment cylindrical, same length as second segment; with seven naked apical setae with claw-like tips; with two spinular rows on outer margin.

***Labrum*** (Fig. 11D) trapezoidal. Anterior surface with two groups of long spinules. Distal margin with lateral outgrowths with small denticles and 11 medial teeth.

***Mandible*** (Fig. 12E) composed of coxa and small palp. Coxal gnathobase with 14 distal elements: one serrate seta; eight strong teeth; five slender teeth, three of these fused in base; gnathobase distally with row of long spinules. Palp with three apical setae: two long and plumose and one short and naked.

***Paragnaths*** (Fig. 11E) with paired lateral lobes. Distal parts of lobes with folded outgrowths. Proximal part of lobes on posterior side with three transverse group of spinules; medial part on posterior side with two longitudinal rows of spinules; distal part with one dense longitudinal posterior, one longitudinal anterior and distal rows of spinules.

Ventral surface of cephalothorax posteriorly to paragnaths with groups of long hairs.

**Figure 13.**
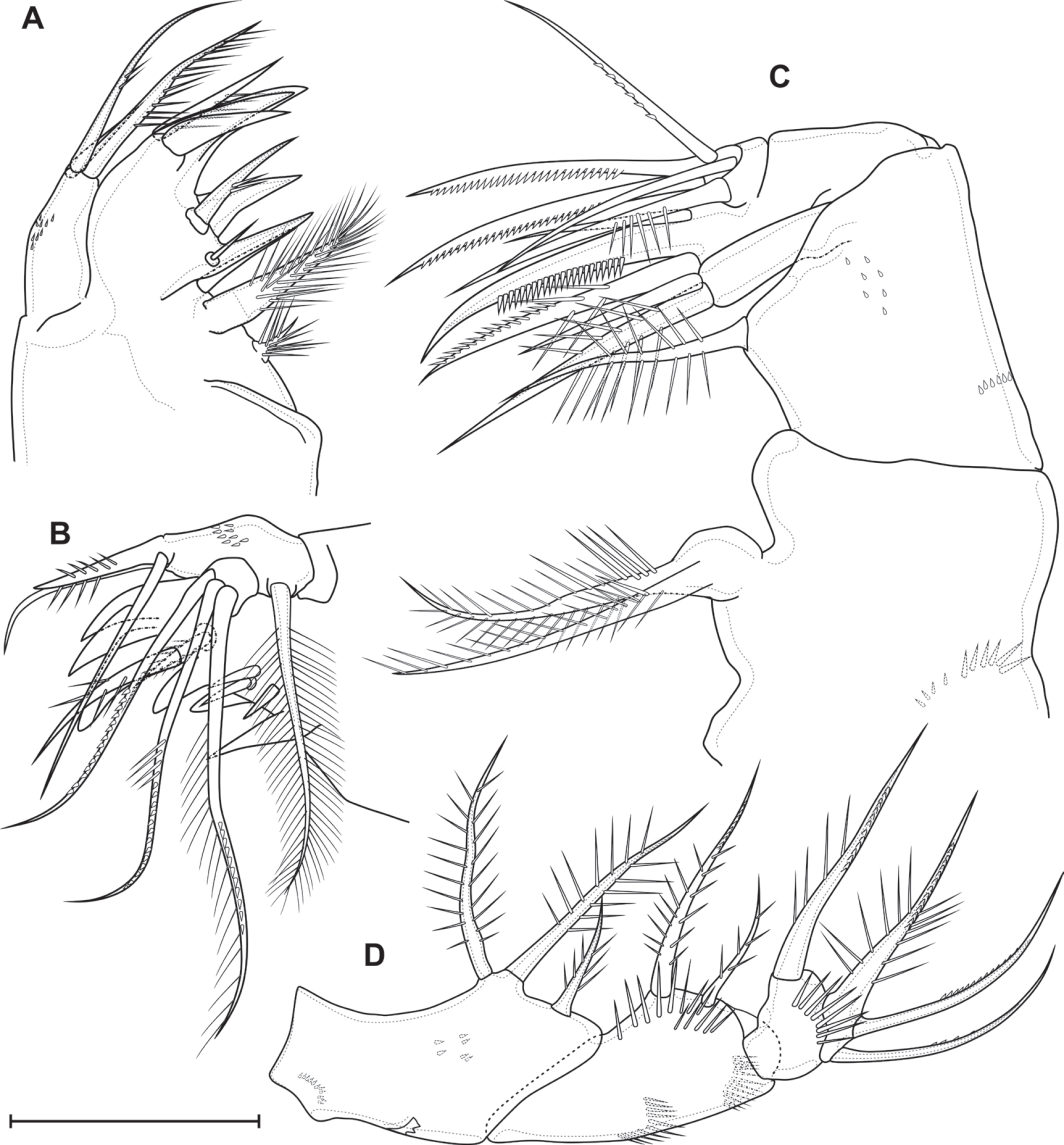
*Eucyclopssibiricus* sp. nov. Female, holotype **A** maxillule, anterior **B** maxillule, posterior **C** maxilla, posterior **D** maxilliped, anterior. Scale bar: 50 μm.

***Maxillule*** (Fig. 13A, B) composed of syncoxa and two-segmented palp: one-segmented basis and one-segmented endopod. Praecoxal arthrite medially with three distal strong teeth, distal pinnate seta, two strong medial spines, three medial setae, one short plumose proximal seta and one long pilose robust proximal seta. Basis with anterior group of spinules; distally with two setae and one spine. Exopod represented by one plumose seta on proximal part of basis. Endopod with three plumose setae.

***Maxilla*** (Fig. 13C) five-segmented, composed of syncoxa, basis and three-segmented endopod. Syncoxa with two rows of spinules on anterior side; with endite with two long pinnate setae. Basis with postero-lateral row of spinules and group of small spinules on posterior side; with two endites: proximal with one plumose seta; distal with one plumose and one bare setae. First endopodal segment with slender seta, strong subdistal plumose seta and strong claw with longitudinal row of spinules. Second endopodal segment with one strong serrate and one strong plumose setae. Third endopodal segment with one seta with small spinules, one bare and one strong serrate seta.

***Maxilliped*** (Fig. 13D) four-segmented, composed of syncoxa, basis and two-segmented endopod. Syncoxa elongated; on anterior side with proximal row of spinules, medial flap-like structure and medial group of small spinules; with three plumose setae. Basis with two outer rows of spinules; two rows of spinules on anterior side; with two plumose setae. First endopodal segment with row of spinules on anterior side and long plumose seta. Second endopodal segment with one plumose and two serrulate setae.

Setae/spine armature of P1 – P4 presented in Table 7.

**Table 7.** P1–P4 armature of *Eucyclopssibiricus* sp. nov.

	Coxa	Basis	Exopod	Endopod
P1	0-1	1-1	I-1; I-1; III, 2, 3	0-1; 0-2; 1, I+1; 3
P2	0-1	1-0	I-1; I-1; III, I+1, 4	0-1; 0-2; 1, I+1; 3
P3	0-1	1-0	I-1; I-1; III, I+1, 4	0-1; 0-2; 1, I+1; 3
P4	0-1	1-0	I-1; I-1; II, I+1, 4	0-1; 0-2; 1, II; 2

**Figure 14.**
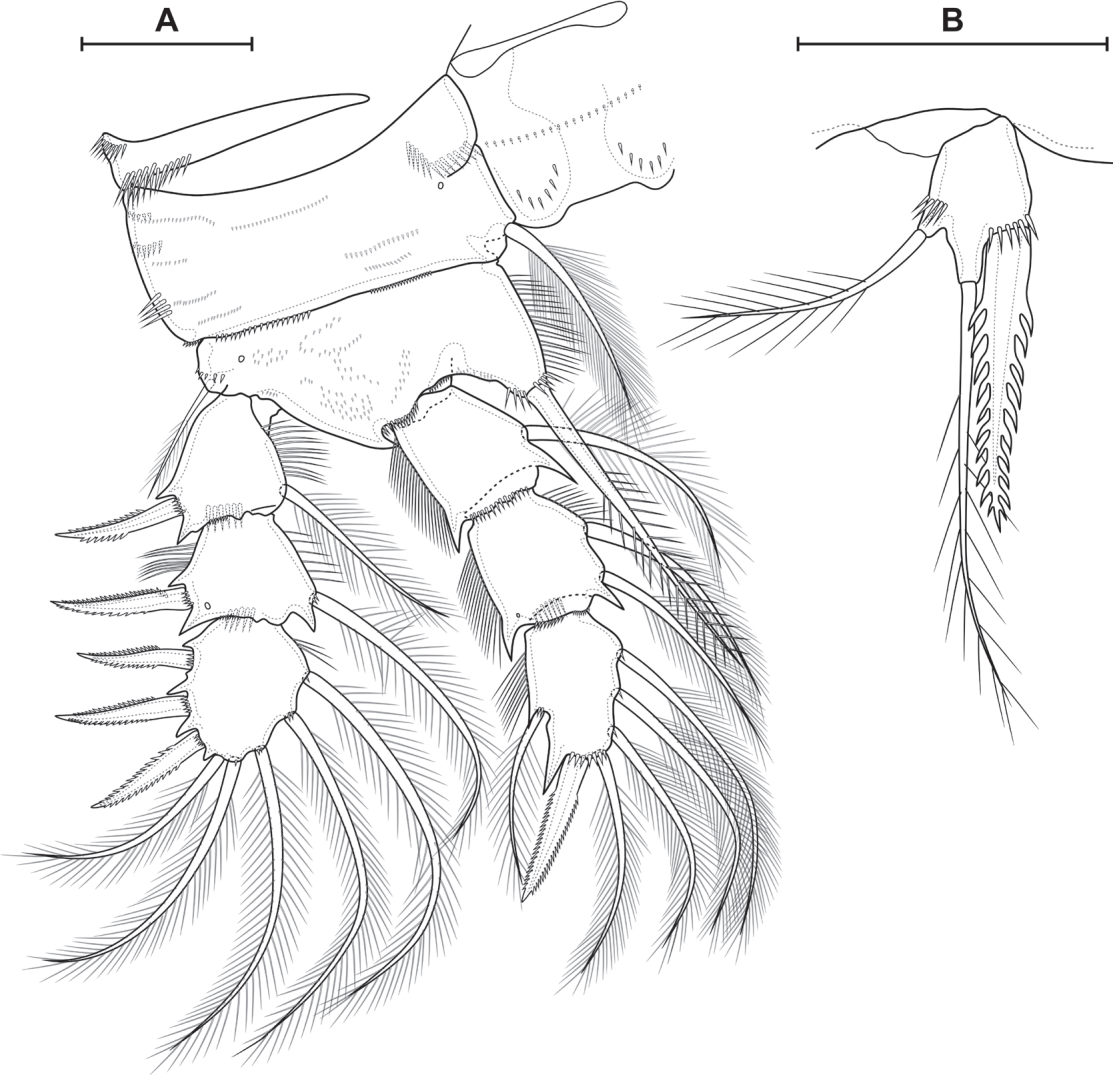
*Eucyclopssibiricus* sp. nov. Female, holotype **A** P1, anterior **B** P5, anterior. Scale bars: 50 μm.

***P1*** (Fig. 14A) with three-segmented rami. Praecoxa with two rows of long spinules. Coxa rectangular; with two posterolateral, one anterolateral and one posteromedial rows of spinules; distal anterior margin with small spinules; anterior side with one pore and seven rows of tiny spinules; with long pinnate inner seta. Intercoxal sclerite almost square, with anterior distal paired and posterior medial continuous rows of spinules. Basis with pore; spinulose rows at base of endopod and inner seta; inner row of setules; outer row of small spinules; large number ungrouped tiny spinules on anterior side; with outer plumose and inner thick pinnate setae. All exopodal and endopodal setae plumose; all exopodal and endopodal spines spinulose. First exopodal segment with outer spine and inner seta; distal posterior, distal anterior rows of spinules, inner row of setules, spinules at base of outer spine. Second exopodal segment with inner seta and outer spine; with anterior pore, distal posterior, distal anterior rows of spinules; inner and outer rows of setules, spinules at base of seta and spine. Third exopodal segment with three outer spines, two apical and three inner setae; with inner setules and spinules at base of setae and spines. First endopodal segment with inner seta; with anterior distal rows of spinules and outer setules. Second endopodal segment with two inner setae; with one pore, distal anterior and distal posterior rows of spinules, outer and inner row of setules. Third endopodal segment with outer seta, apical spine, apical seta, three inner setae; with outer row of setules and spinules at base of spine and setae.

**Figure 15.**
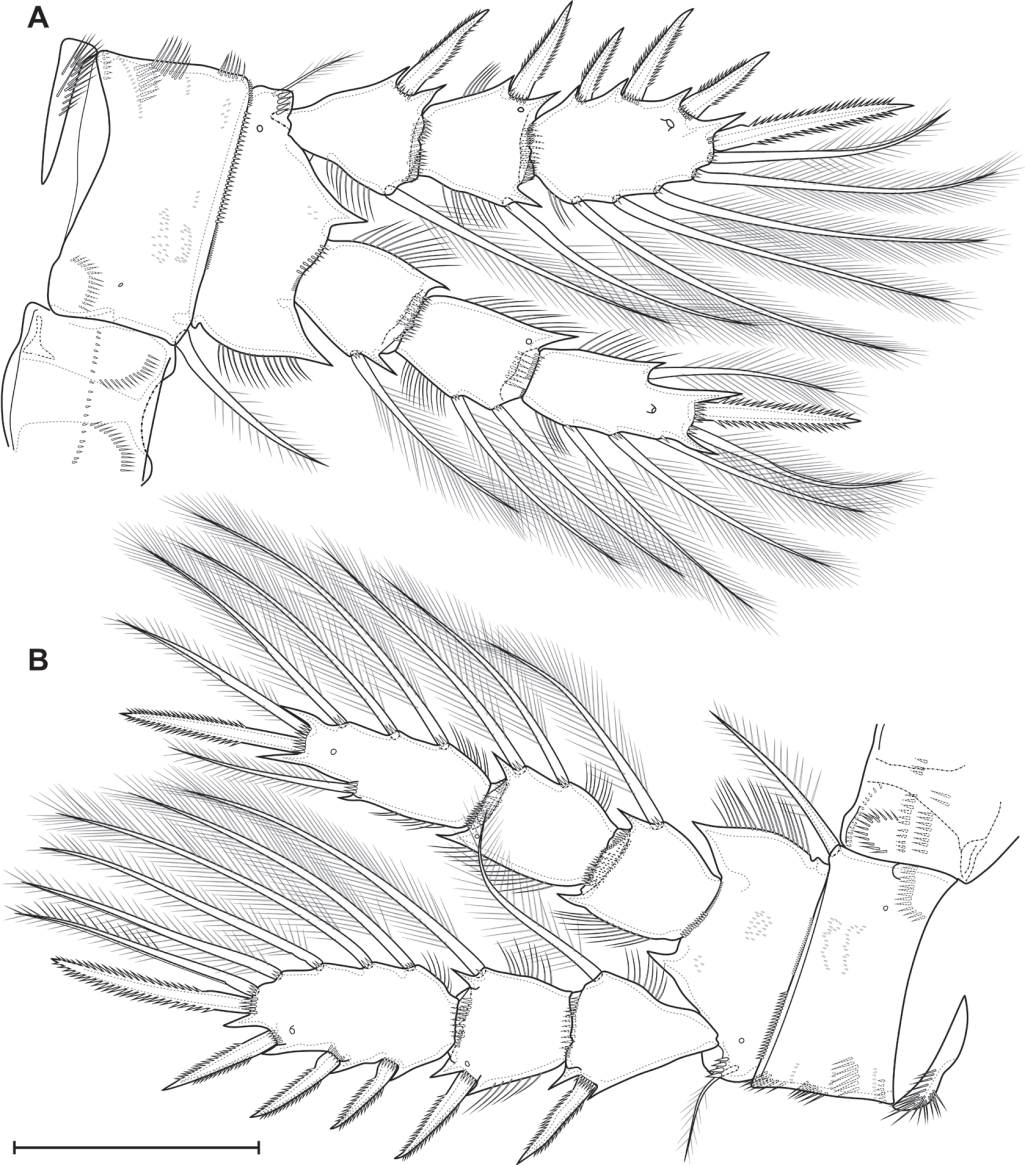
*Eucyclopssibiricus* sp. nov. Female, holotype **A** P2, anterior **B** P3, anterior. Scale bar: 100 μm.

***P2*** (Fig. 15A) with three-segmented rami. Praecoxa with group of long spinules. Coxa rectangular; with two posterolateral, two anterolateral and one posteromedial row of spinules; distal anterior margin with small spinules; anterior side with one pore and six rows of tiny spinules; with pinnate inner seta. Intercoxal sclerite almost square, with anterior distal paired and posterior medial continuous rows of spinules. Basis with pore; spinulose rows at base of endopod and on outer side; inner row of setules; several ungrouped tiny spinules on anterior side; with outer plumose seta. All exopodal and endopodal setae plumose; all exopodal and endopodal spines spinulose. Segments with small spinules near base of all spines and setae, except inner seta of first exopodal segment. First exopodal segment with outer spine and inner seta; distal posterior, distal anterior rows of spinules, inner row of setules. Second exopodal segment with inner seta and outer spine; with anterior pore, distal posterior, distal anterior rows of spinules; inner and outer rows of setules. Third exopodal segment with three outer spines, one apical spine, one apical seta and four inner setae; with one pore and inner setules. First endopodal segment with inner seta; with distal anterior and distal posterior rows of spinules and outer row of setules. Second segment with two inner setae; with one pore, distal anterior and distal posterior rows of spinules, and outer and inner rows of setules. Third segment with one outer seta, one apical spine, one apical seta, and three inner setae; with one pore; with outer and inner row of setules.

***P3*** (Fig. 15B) similar to P2. Intercoxal sclerite on posterior side with two rows of spinules.

**Figure 16.**
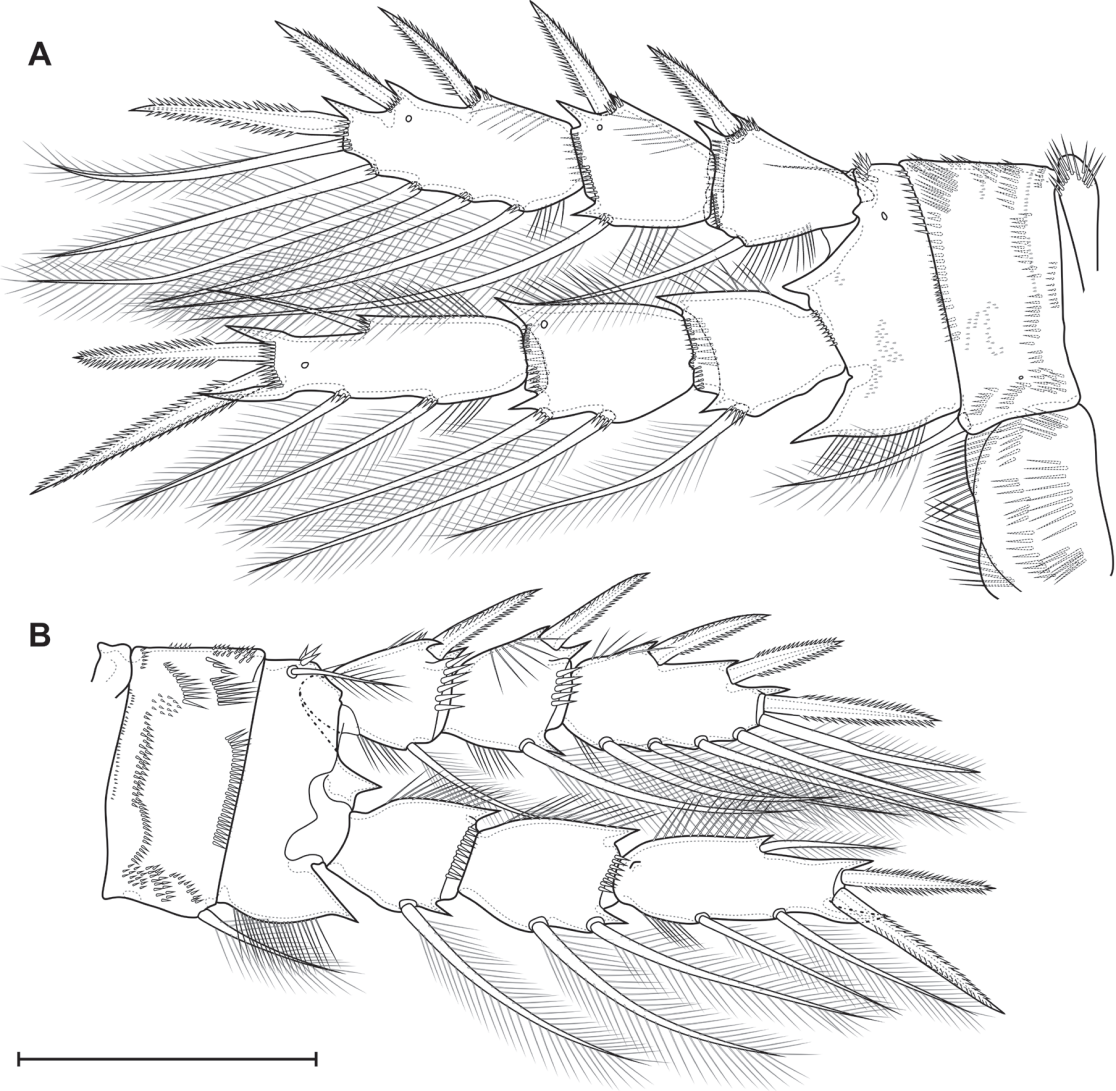
*Eucyclopssibiricus* sp. nov. Female **A** holotype, P4, anterior **B** var. brevis, P4, posterior. Scale bar: 100 μm.

***P4*** (Fig. 16A, B) with three-segmented rami. Praecoxa with group of long spinules. Coxa rectangular; with pinnate inner seta. Posterior side of coxa with three proximal straight rows, three medial groups, several lateral groups and rows, and one distal row of large spinules. Anterior side with one pore and six rows of tiny spinules; with spinules on distal margin. Intercoxal sclerite on posterior side with continuous distal row of setules and discontinuous medial and proximal rows of long spinules/setules. Basis with pore; spinulose rows at base of endopod and on outer side; inner row of setules; several ungrouped tiny spinules on anterior side; with outer plumose seta. All exopodal and endopodal setae plumose; all exopodal and endopodal spines spinulose. Segments with small spinules near base of all spines and setae, except inner seta of first exopodal segment. First exopodal segment with outer spine and inner seta; distal posterior, distal anterior rows of spinules; inner row of setules; number of inner setules 8–17 (median 12, *n* = 13). Second exopodal segment with inner seta and outer spine; with anterior pore, distal posterior, distal anterior rows of spinules; inner and outer rows of setules; number of inner setules 6–10 (median 7, *n* = 13). Third exopodal segment with two outer spines, one apical spine, apical seta, and four inner setae; with one pore, outer and inner setules; three distal setae with hyaline frill. First endopodal segment with inner seta; with distal anterior and distal posterior rows of spinules and outer row of setules. Second segment with two inner setae; with one pore, distal anterior and distal posterior rows of spinules, outer row of setules. Third segment with one outer and one apical seta, one apical spine, and three inner setae; with one pore; with outer and inner row of setules; with small proximal outgrowth; all setae with weakly expressed hyaline frill.

***P5*** (Fig. 14B) one-segmented, without intercoxal sclerite. Segment with large robust inner spine, one apical pinnate and outer pinnate setae; with row of spinules near base of inner spine and with or without outer row of spinules.

**Male.** Significantly smaller than female (Fig. 9E–G). Total body length from anterior margin of rostrum to posterior margin of caudal rami: 870 µm (*n* = 1), largest width of cephalothorax 255 µm (*n* = 1). Sexual dimorphism expressed in the antennule, ornamentation of A2, P2–P4, genital segmentation and ornamentation, P6, caudal rami. Pore pattern of cephalothorax and abdominal somites as in female.

**Figure 17.**
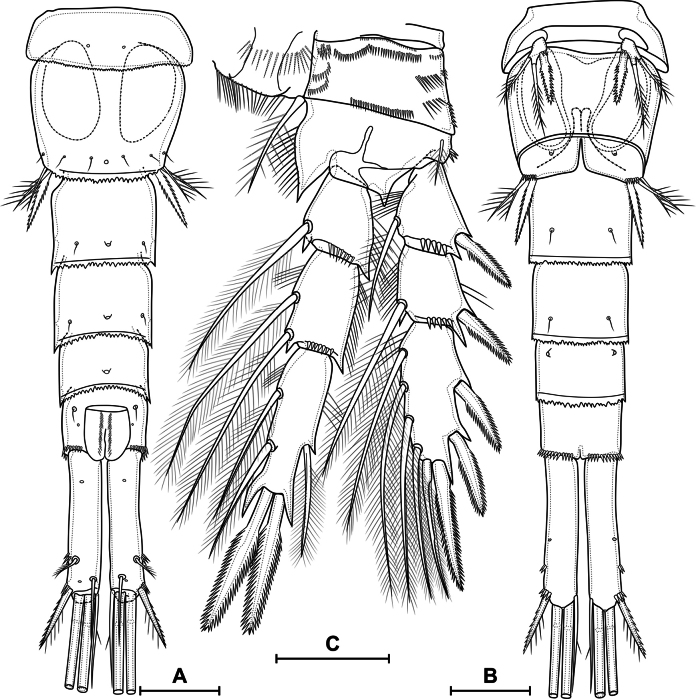
*Eucyclopssibiricus* sp. nov. Male, paratype **A** urosome, dorsal **B** urosome, ventral **C** P4, posterior. Scale bars: 50 μm.

***Genital somite*** (Fig. 17A, B) free; with two spermatophores visible inside; with P6. Spermatophores (Fig. 17B) bean-shaped. P6 (Fig. 17B) marked by two large flaps fused to the somite; with ventral pores; with outer and middle pinnate setae and inner long spinulose spine.

**Figure 18.**
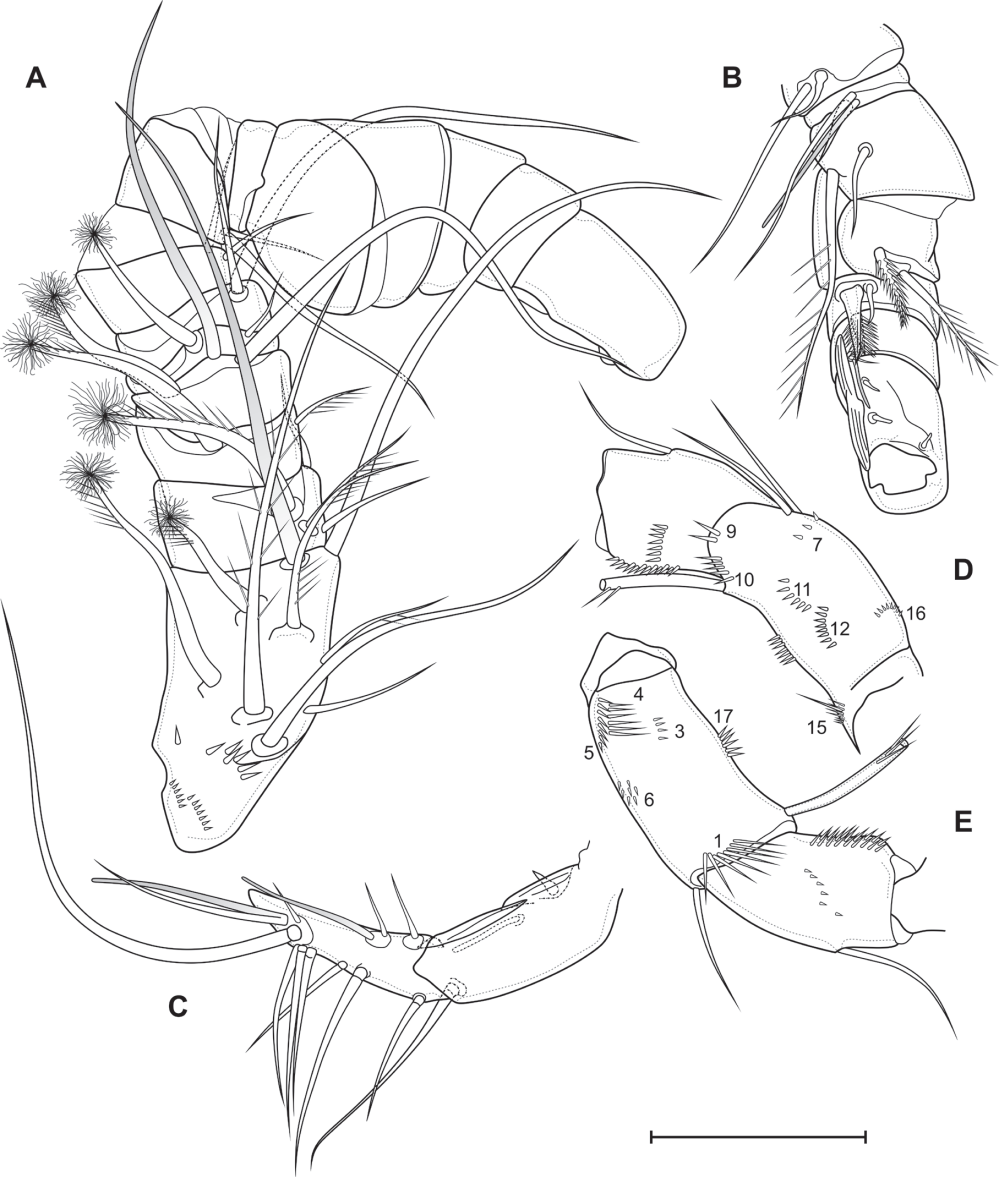
*Eucyclopssibiricus* sp. nov. Male, paratype **A** antennule, segments 1–14, setae on segments 9–14 not figured **B** antennule, segments 8–14 **C** antennule, segments 15–16 **D** coxobasis and first endopodal segment of antenna, anterior **E** coxobasis and first endopodal segment of antenna, posterior. Scale bar: 50 μm.

***Antennule*** (Fig. 18A–C) 16-segmented, haplocer with two geniculations: first one between segments 7, 8, and 9; second one between segments 14 and 15. Segment 1 long; with three spinulose rows; one long setiform aesthetasc, six unmodified setae and two modified “blowball-setae” with abruptly breaking tip with dense bunch of setules. Segment 2 with three unmodified setae and one “blowball-seta”. Segment 3 with one unmodified seta and one “blowball-seta”. Segment 4 with one long setiform aesthetasc, one unmodified seta and one “blowball-seta”. Segment 5 with one unmodified seta and one “blowball-seta”. Segments 6, 7, 8 similar, short, each with two setae. Segment 9 with two unmodified setae and one aesthetasc. Segments 10 and 11 widened, each with two setae. Segment 12 with one small and one spiniform robust seta. Segment 13 with one robust serrate seta, one small seta and one aesthetasc. Segment 14 elongated, with one modified flattened laminar seta and three small finger-like modified setae. Segment 15 elongate; with two modified flattened setae; one long unmodified seta; one modified spiniform seta and one aesthetasc. Segment 16 with two aesthetascs, eleven setae, one of which fused in base with aesthetasc. Armature formula: 1-[8+ae],2-[4],3-[2],4-[2+ae],5-[2],6-[2],7-[2],8-[2],9-[2+ae],10-[2],11-[2],12-[2],13-[2+ae],14-[4],15-[4+ae],16-[10+ae+(1+ae)].

***Antenna*** (Fig. 18D, E). Endopod similar to that in female. Coxobasis rectangular; with three setae, one of these exopodal long pinnate seta; with groups of spinules 1, 3, 4, 5, 6, 7, 9, 10, 11, 12, 15, 16.

***P2–P4*** (Fig. 17C) similar to this in female, differences in relative length and shape of segments; smaller ornamentation; spines longer with strong spinules; setae shorter, with well-expressed hyaline frills.

##### Variability.

Eucyclopssibiricussp. nov.var.brevis does not differ in the structure of the caudal rami (Fig. 11A) and coxobasis of the antennae (Fig. 12C, D). The differences concern only the relative length of the P2–P4 segments (Fig. 16B).

#### ﻿ Eucyclops speratus

Taxon classificationAnimaliaCyclopoidaCyclopidae

(Lilljeborg, 1901)

97A5F10F-C10A-5745-A0CF-9C0733A6F3A7

Figs 19 , 20 , 21

Cyclops varius var. speratus Lilljeborg 1901: 88, tab. V: figs 12–15.
Leptocyclops speratus : Sars 1914: 72, pl. XLIV.
Cyclops agilis speratus : Gurney 1933: 104, figs 1395–1405.
Eucyclops speratus : Monchenko 1974: 91, fig. 40; Dumont and Decraemer 1977: 262, figs 3C, 4; Ishida and Hiruta 1999 (part): 88, fig. 2f–j; Alekseev et al. 2006: 136, fig. 14.
Eucyclops (Speratocyclops) speratus : Alekseev 2019: 511, fig. 13.

##### Material examined.

Russia • 1 ♀ dissected on two slides; Saint Petersburg, Orlov Pond; 59.84890°N, 30.04384°E; 20 June 2023; E. Chertoprud leg; EMKSU VH 2000/21–22 (N° 86); • 2 ♀ dissected on two slides; Saint Petersburg, Orlov Pond; 59.84890°N, 30.04384°E; 20 June 2023; E. Chertoprud leg; in collection of A. Novikov (N° 98, 111); • 1 ♀ dissected on one slide; Saint Petersburg, Olginsky Pond; 60.01293°N, 30.35514°E; 20 June 2023; E. Chertoprud leg; in collection of A. Novikov (N° 97); • 3 ♀ dissected on three slides; Tatarstan republic, Naberezhnie Morkvashi village, puddle on stream; 55.76324°N, 48.85700°E; 28 June 2020; A. Novikov and D. Sharafutdinova leg; in collection of A. Novikov (N° 75, 103, 104); • 6 ♀ dissected on six slides; Udmurtia republic, Chistostem village, pond; 56.86162°N, 52.21630°E; 16 June 2018; A. Novikov leg; in collection of A. Novikov (N° 30, 59, 60, 93, 95, 96)

##### Description.

**Figure 19.**
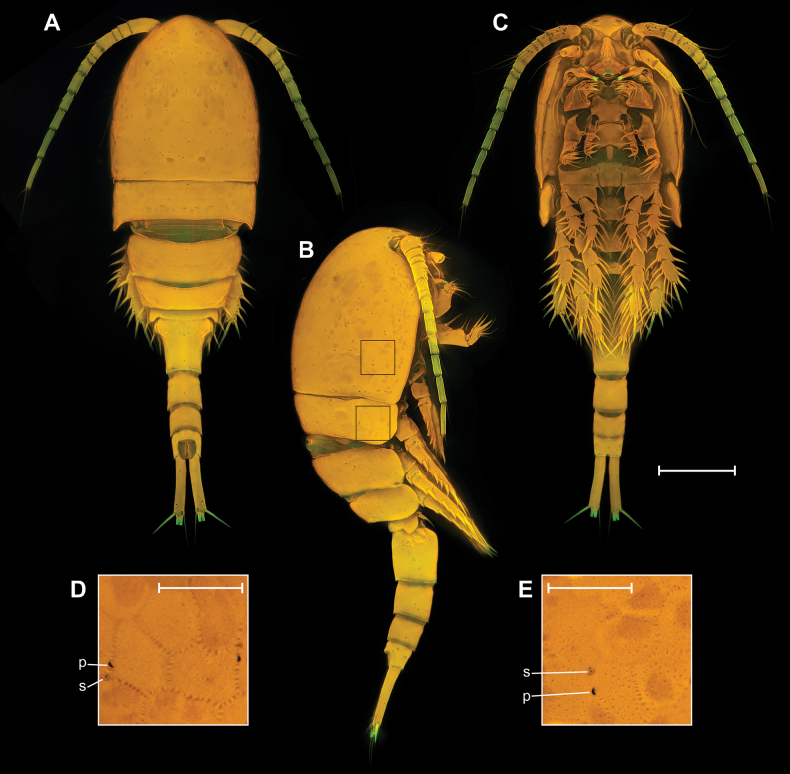
*Eucyclopssperatus*. Female (Udmurtia), habitus: **A** dorsal **B** lateral **C** ventral. Ornamentation of somites: **D** second pedigerous somite **E** cephalothorax. Abbreviations: s – sensillum, p – pore. Scale bars: 200 μm (**A–C**); 50 μm (**D, E**).

**Figure 20.**
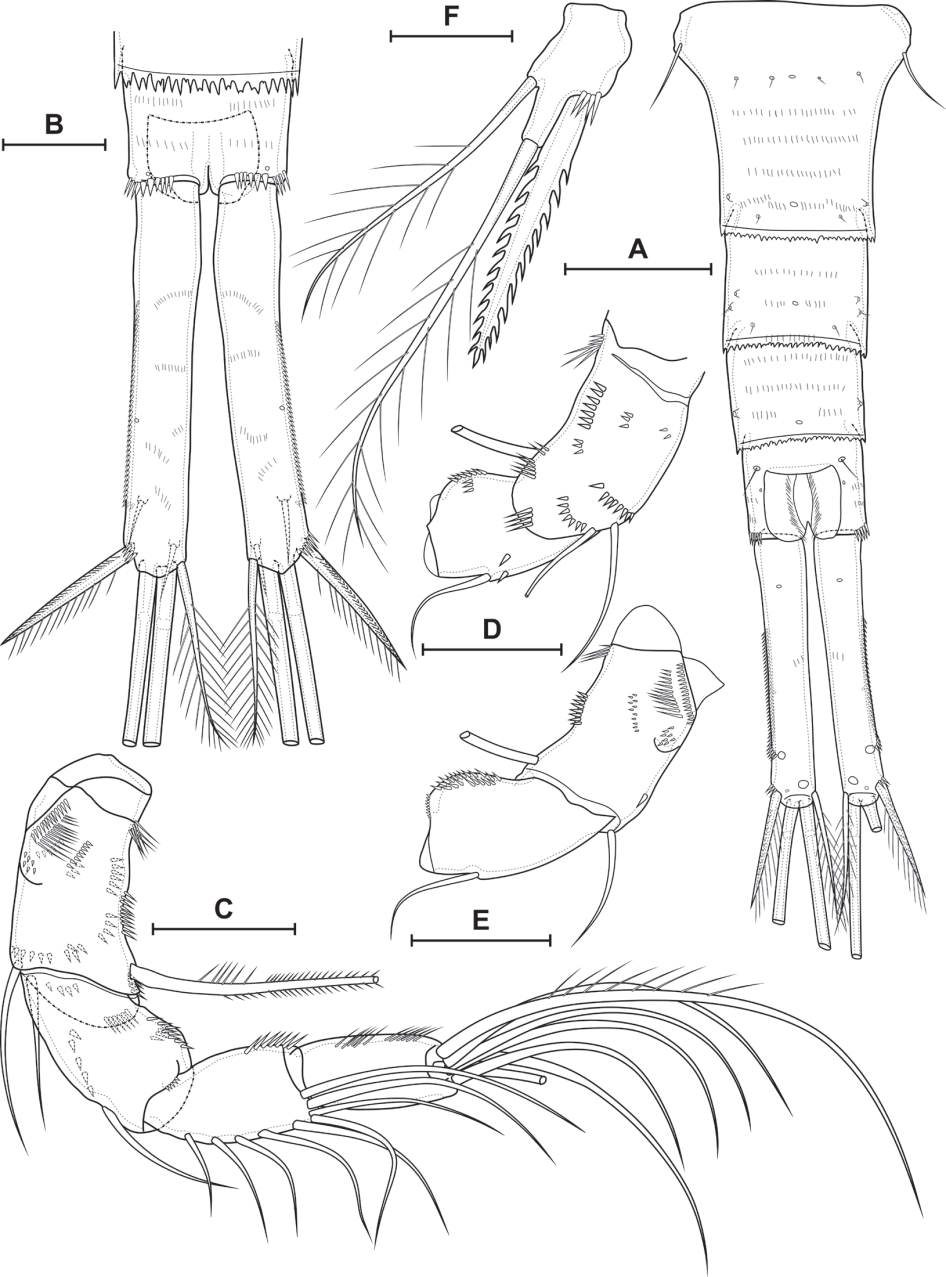
*Eucyclopssperatus*. Female **A** abdomen, dorsal (Saint-Petersburg) **B** caudal rami, ventral (Udmurtia) **C** antenna, posterior (Saint-Petersburg) **D** coxobasis and first endopodal segment of antenna, anterior (Udmurtia) **E** coxobasis and first endopodal segment of antenna, posterior (Udmurtia) **F** P5, anterior (Saint-Petersburg). Scale bars: 100 μm (**A**); 50 μm (**B–E**); 20 μm (**F**).

**Female. *Body elongated*** (Fig. 19A–C). Total body length from anterior margin of rostrum to posterior margin of caudal rami: mean 1310 µm (*n* = 1). Cephalothorax pitted (Fig. 19E), wider than first and second free somites, largest width 395 µm (*n* = 1), surface with 253 pores and sensilla, with all groups (Fig. 8A–D). Naupliar eye not observed. Posterior margin of cephalothorax and all pedigerous somites smooth. Pedigerous and abdominal somites pitted, with “sutures” (Figs 19D, 20A, B).

***Abdomen*** and ***P6*** similar to that in *E.sibiricus* sp. nov. (Fig. 20A).

***Caudal rami*** similar to that in *E.sibiricus* sp. nov. (Fig. 20A, B). Length/width ratio 5.4–6.3 (median 6.04, *n* = 13). Serra extending from base of seta II to anterior 20% of ramus (10%–36%, median 20%, *n* = 13). Ratio of length of seta VI to seta III 1.07–1.28 (median 1.18, *n* = 13).

Antennules, antennae, mouthparts, labrum, and paragnaths similar to that in *E.sibiricus* sp. nov.

***Antenna*** (Fig. 20C–E). Coxobasis with groups of spinules 3, 4, 5, 6, 7, 8, 9, 10, 11, 12, 14, 15, 16. Number of spinules of group 7: 3–10 (median 5, *n* = 13). Number of spinules of group 8: 0–10 (median 4, *n* = 13).

**Figure 21.**
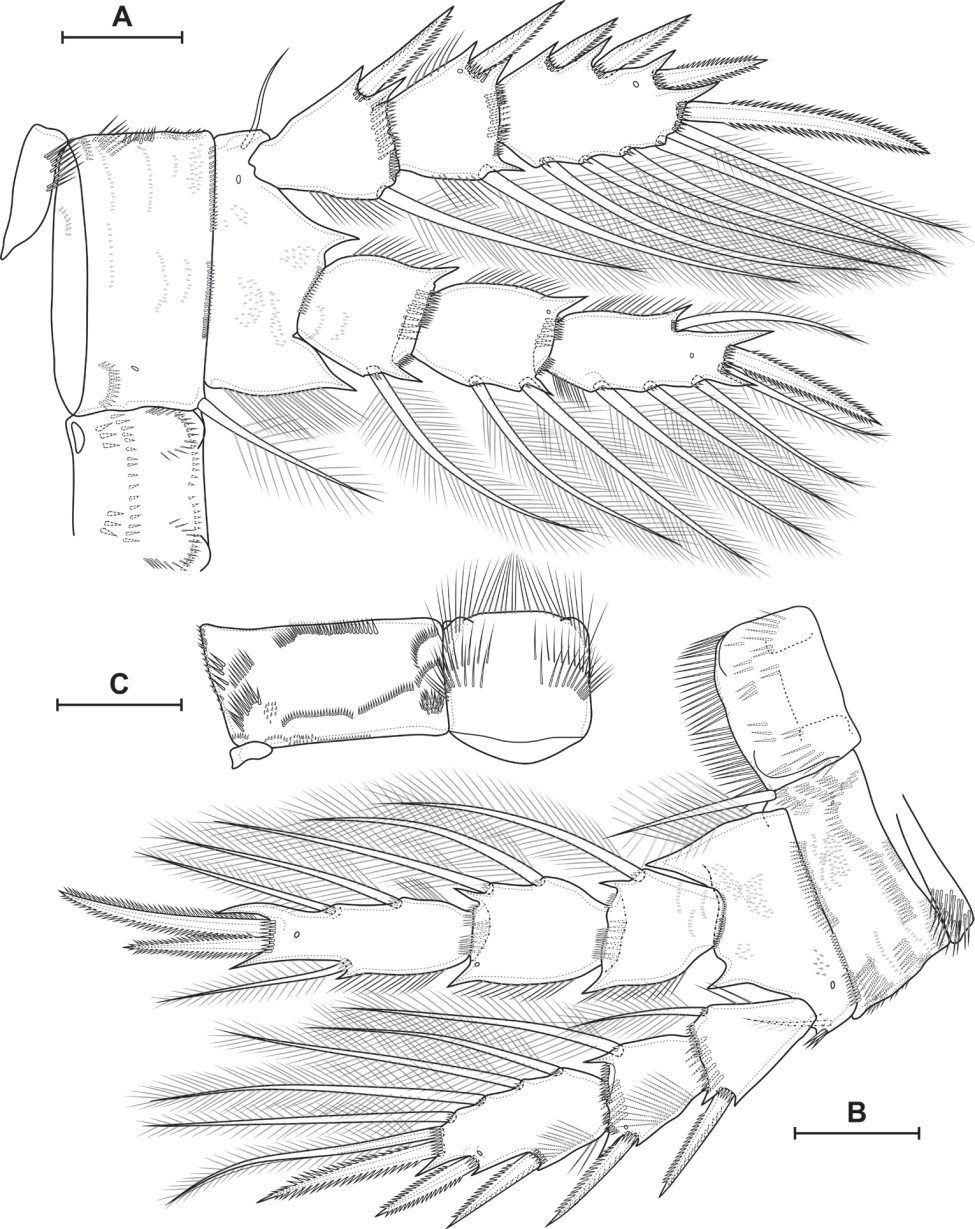
*Eucyclopssperatus*. Female **A** P3, anterior (Saint-Petersburg) **B** P4, anterior (Saint-Petersburg) **C** P4 coxa and intercoxal sclerite, posterior (Udmurtia). Scale bars: 50 μm.

***P1–P4*** similar to that in *E.sibiricus* sp. nov., but with shorter segments (Fig. 21A–C). Number of inner setules of P4 Exp1 (Fig. 21B): 1–6 (median 1, *n* = 13). Number of inner setules of P4 Exp2: 1–3 (median 0, *n* = 13).

***P5*** (Fig. 20F) similar to that in *E.sibiricus* sp. nov., but without outer row of spinules.

**Male.** Not studied.

## ﻿Discussion

### ﻿*Eucyclopssperatus* species complex

The phylogenetic relationships of *E.speratus* are unclear. According to Alekseev (2019), it belongs to the subgenus E. (Speratocyclops) together with the species group *E.arcanus* Alekseev, 1990. In our opinion, it is currently impossible to construct a phylogeny of the genus. Morphological data on most species of the genus are very incomplete. Genetic methods give an ambiguous picture with a small sample of species. Therefore, we believe that Alekseev’s allocation of subgenera was premature. Some of its subgenera do not have synapomorphies. For example, Eucyclops (Eucyclops) is distinguished by the presence of groups 1 and 2 of the antenna coxobasis (Alekseev 2019). However, this is a plesiomorphic character, present, for example, in females of the *E.macruroides* species group (unpublished data) and in males of *E.sibiricus* sp. nov. The presence of a gap on the coxal seta P4 is quite variable, for example, it is sometimes absent in *E.roseus* and *E.azorensis* (Hołyńska et al. 2021). Therefore, in this study, we deliberately do not use subgenera taxonomy, due to too few data for a full re-assessment of the subgenera.

The diagnostic features of *E.speratus* in our understanding are antenna coxobasis with group 9 and without groups 1 and 2; maxillular palp with a group of spinules; second segments of P1–P4 exopods with outer setules, proctodeum with setules. The same set is possessed by *E.sibiricus* sp. nov. Without the use of molecular genetic methods, it would be unlikely to detect this species due to extremely weak differences from *E.speratus*. We believe that the only reliable character for separating these two species is the setules on the inner side of P4 Exp1–Exp2. The remaining characters are largely variable and overlap in these two species.

In some keys, a shortening of the serra of the caudal rami is indicated as a characteristic of *E.speratus* (Monchenko 1974). However, as Ishida showed, this character is not found in Western European populations (Ishida and Hiruta 1999). According to our data, the length of the serra varies greatly even within individuals from the same waterbody.

### ﻿The taxonomic status of *E.sibiricus* sp. nov.

Although *E.sibiricus* sp. nov. and *E.speratus* form one cluster on the phylogenetic trees, based on two molecular markers, the COI genetic distances between *E.sibiricus* sp. nov. and *E.speratus* from the European part of Russia (11.4–14.4%) correspond to the level of interspecific distances known for Cyclopida and Copepoda in general (Krajíček et al. 2016; Sukhikh et al. 2023). Additionally, two delimitation methods based on different approaches yielded identical division into species, where *E.sibiricus* sp. nov. and *E.speratus* from the St. Petersburg population belong to two separate species. ITSn of nuclear DNA evolves more slowly than COI, however, even for this fragment, *E.sibiricus* sp. nov. individuals are genetically isolated from *E.speratus* from the European part of Russia. Additionally, the full length of ITS1 in two representatives of *E.sibiricus* sp. nov. is identical (391 bp) and differs from that of the representative of *E.speratus* (388 bp) from St. Petersburg and may be a species-specific character, as shown for *Diacyclops* Kiefer, 1927 from Lake Baikal and the genus *Culicoides* Latreille, 1809 (Diptera) (Li et al. 2003; Mayor et al. 2024a). *Eucyclopssibiricus* sp. nov. is genetically heterogeneous. Two phylogroups are distinguished based on the fastest evolving gene COI, one of which is represented by individuals from the Putorana Plateau and the second is represented by individuals from the Lena River Delta and Irkutsk region. The distribution of the second group may reflect the dispersal route of the species in connection with the Lena River, the source of which begins in the Irkutsk region. The genetically closest representatives to *E.sibiricus* sp. nov. are *E.speratus* individuals found in the Irkutsk region and on Sakhalin Island, whose sequences are publicly available in GenBank (Mayor et al. 2024b). Also, unpublished sequences with high similarity were found in BOLD from *E.speratus* individuals from the Irkutsk region. Probably, these individuals are also representatives of *E.sibiricus* sp. nov. and were identified by the authors as *E.speratus* due to the similar morphology of the two species. This assumption is confirmed by the results of species delimitation conducted by two methods. The ITSn fragment showed the greatest genetic diversity among *E.speratus* individuals from Sakhalin Island. All three individuals united into a phylogroup with *E.sibiricus* sp. nov., but one individual is genetically divergent and forms a separate genetic line, also distant from *E.speratus* from the European part of Russia. Genetic heterogeneity among *E.speratus* according to the conservative fragment of 18S rRNA among individuals from distant populations (European part of Russia and Korea) also indicates the presence of a species complex among *E.speratus*.

In general, *E.speratus* and *E.sibiricus* sp. nov. can be considered pseudocryptic species (Lajus et al. 2015). The morphological differences between them would be insufficient for separation without the use of molecular genetic methods. At the same time, they cannot be called subspecies of the same species, since their ranges overlap (particularly in Tatarstan) and there are no intermediate forms. Weak morphological differences between the species can be explained by allopatric speciation. Initially isolated species occupied identical ecological niches, so rapid molecular evolution was accompanied by morphological stasis (Fišer et al. 2018).

Linear morphometry has proven to be of little use for identifying pseudocryptic *Eucyclops* species. The same is shown for the Baikalian Copepoda*Harpacticellainopinata* Sars G.O., 1908 species complex (Kochanova et al. 2024) and *Diacyclops* (Mayor et al. 2024a). Geometric morphometry based on segment shape and integumental organ patterning has shown little utility, although these characters have shown high utility in other Copepoda species complexes (Karanovic and Kim 2014; Karanovic and Bláha 2019; Karanovic 2022). Quantitative microcharacters have shown the greatest efficiency, higher, for example, than that of the genus *Acanthocyclops* Kiefer, 1927 (Karanovic and Bláha 2019). For different genera there are specific characters and methods that can separate closely related species. The shape of the P4 segments is suitable for separating the *Acanthocyclopsvernalis* (Fischer, 1853) species complex, but not for the *E.speratus* species complex. As recommendations, we can say that when describing pseudocryptic species, it is highly desirable to use a set of at least several methods and be sure to attach the results obtained for each individual for the possibility of subsequent use.

### ﻿Taxonomic status of previous *E.speratus* records in the literature

The Korean (Lee et al. 2005) and Japanese (Ishida and Hiruta 1999) individuals probably belong to *E.sibiricus* sp. nov. The Korean individuals have many setules on the inner side of P4 Exp1 and Exp2, a relatively short outer seta on P4 Enp3, and a relatively small number of spinules of groups 7 and 8 of the antennal coxobasis (Lee et al. 2005). The descriptions of the Japanese individuals do not provide illustrations of the P4, only of its Exp3. This segment is shortened, as in E.sibiricussp. nov.var.brevis. Group 8 of the antennal coxobasis is missing in the figure, and group 7 is represented by only two spinules (Ishida and Hiruta 1999), a character of *E.sibiricus* sp. nov.

Some early descriptions are difficult to assign to any species. Ishida (1998) provided figures of the caudal rami and the intercoxal sclerite of the P4 of *E.speratus* from Alaska, Kamchatka, and Japan. However, there is insufficient information for an unambiguous identification. *Eucyclopssperatusifniensis* Dumont & Decraemer, 1977 is probably a synonym of *E.speratus*. The distinguishing character of the subspecies is the group of spinules on the caudal rami (Dumont and Decraemer 1977), which are probably the sutures that are almost always present on the somites and caudal rami of *E.speratus* (Figs 19, 20). The only difference is that the outer seta of the P4 Enp3 is short, like in *E.sibiricus* sp. nov. Therefore, to clarify the taxonomic status, it is necessary to study the type material or individuals from the type locality.

## ﻿Conclusions

The study revealed one more scientifically new Cyclopoida species from Middle Siberia. *Eucyclopssibiricus* sp. nov. shows a wide geographical range extending from the central part of the East European Plain to the Far East, where it is recorded according to literature data. Characteristically, this species was previously mixed with the closely related *E.speratus* on the basis of morphological similarity and was unambiguously identified only due to molecular-genetic analyses of several genes. Prospects for increasing the knowledge of the fauna of Middle Siberia are also seen for some other genera of Cyclopoida, with records of undescribed species known from the Lena River Delta and the Putorana and Anabar plateaus (Chertoprud et al. 2022). Furthermore, performing integrative descriptions will permit discrimination of cryptic taxa and provide an opportunity to clarify the real distribution of Cyclopoida species with wide Palearctic and Holarctic ranges. In addition, comparative analyses of haplotypes from geographically separated populations of species will create prerequisites for identifying the ways of the freshwater Copepoda fauna formation in northeastern Eurasia as a whole.

## Supplementary Material

XML Treatment for Eucyclops sibiricus

XML Treatment for Eucyclops speratus

## ﻿Additional information

### Author ORCIDs

Aleksandr Novikov https://orcid.org/0000-0003-2026-2510

Dayana Sharafutdinova https://orcid.org/0000-0001-6533-5686

Tatyana Mayor https://orcid.org/0000-0003-4425-1330

Elena Chertoprud https://orcid.org/0000-0002-9874-1610

### Data availability

All of the data that support the findings of this study are available in the main text or Supplementary Information.

## Supplementary materials

Supplementary material 1 Data on quantitative characters Data type xlsx Explanation note Data on quantitative characters (number of spinules and setules in groups of spinules), linear morphometrics, landmark-based geometric morphometrics in Procrustes coordinates and integumental pore pattern of the cephalothorax of studied Eucyclops individuals. https://binary.pensoft.net/file/1244445This dataset is made available under the Open Database License (http://opendatacommons.org/licenses/odbl/1.0/). The Open Database License (ODbL) is a license agreement intended to allow users to freely share, modify, and use this Dataset while maintaining this same freedom for others, provided that the original source and author(s) are credited.Aleksandr Novikov, Dayana Sharafutdinova, Ekaterina Abramova, Tatyana Mayor, Elena Chertoprud

Supplementary material 2 Landmark-based geometric morphometrics primary coordinate data for .tps files Data type xlsx https://binary.pensoft.net/file/1244446This dataset is made available under the Open Database License (http://opendatacommons.org/licenses/odbl/1.0/). The Open Database License (ODbL) is a license agreement intended to allow users to freely share, modify, and use this Dataset while maintaining this same freedom for others, provided that the original source and author(s) are credited.Aleksandr Novikov, Dayana Sharafutdinova, Ekaterina Abramova, Tatyana Mayor, Elena Chertoprud
